# *De Novo* Transcriptomic Analysis of Peripheral Blood Lymphocytes from the Chinese Goose: Gene Discovery and Immune System Pathway Description

**DOI:** 10.1371/journal.pone.0121015

**Published:** 2015-03-27

**Authors:** Mansoor Tariq, Rong Chen, Hongyu Yuan, Yanjie Liu, Yanan Wu, Junya Wang, Chun Xia

**Affiliations:** 1 Department of Microbiology and Immunology, College of Veterinary Medicine, China Agricultural University, Beijing, The People’s Republic of China; 2 Department of Veterinary Pathology, Faculty of Animal Husbandry and Veterinary Sciences, Sindh Agriculture University, Tando Jam, Sindh, Pakistan; 3 Key Laboratory Zoonosis of Ministry of Agriculture of China, Beijing, The People’s Republic of China; Chinese Academy of Sciences, CHINA

## Abstract

**Background:**

The Chinese goose is one of the most economically important poultry birds and is a natural reservoir for many avian viruses. However, the nature and regulation of the innate and adaptive immune systems of this waterfowl species are not completely understood due to limited information on the goose genome. Recently, transcriptome sequencing technology was applied in the genomic studies focused on novel gene discovery. Thus, this study described the transcriptome of the goose peripheral blood lymphocytes to identify immunity relevant genes.

**Principal Findings:**

*De novo* transcriptome assembly of the goose peripheral blood lymphocytes was sequenced by Illumina-Solexa technology. In total, 211,198 unigenes were assembled from the 69.36 million cleaned reads. The average length, N50 size and the maximum length of the assembled unigenes were 687 bp, 1,298 bp and 18,992 bp, respectively. A total of 36,854 unigenes showed similarity by BLAST search against the NCBI non-redundant (Nr) protein database. For functional classification, 163,161 unigenes were comprised of three Gene Ontology (Go) categories and 67 subcategories. A total of 15,334 unigenes were annotated into 25 eukaryotic orthologous groups (KOGs) categories. Kyoto Encyclopedia of Genes and Genomes (KEGG) database annotated 39,585 unigenes into six biological functional groups and 308 pathways. Among the 2,757 unigenes that participated in the 15 immune system KEGG pathways, 125 of the most important immune relevant genes were summarized and analyzed by STRING analysis to identify gene interactions and relationships. Moreover, 10 genes were confirmed by PCR and analyzed. Of these 125 unigenes, 109 unigenes, approximately 87%, were not previously identified in the goose.

**Conclusion:**

This *de novo* transcriptome analysis could provide important Chinese goose sequence information and highlights the value of new gene discovery, pathways investigation and immune system gene identification, and comparison with other avian species as useful tools to understand the goose immune system.

## Introduction

Genomic evaluations are the source of important evidence for determining the immune system characteristics that differ between the goose, chicken, duck, and other birds. After genome sequencing of the chicken and duck, both became the first-class avian species, which allows immunologic comparison with other animals [1,2]. Subsequently, the avian species have been further elucidated in the evolutionary process [2], with the recent availability in fish and mammal genomes [3]. The goose genome requires study because it is one of the most important waterfowl species and is also a vital component in the fast-growing poultry economy of China, which has become the largest goose production country in the world [2].

Interest in the goose immune system comes not only from its importance as a food animal species, but also for its role as a natural reservoir of many avian viruses, such as influenza virus [4]. Therefore, it is essential to illustrate the nature and regulation of the innate and adaptive immune systems in the goose [5]. However, except chicken and other poultry such as goose, relatively little is known about their immune systems at the molecular level [6]. Thus, the discovery of important immune genes and functional studies can help elucidate immunological responses and the natural or inherited disease resistance ability. To date, only a few research studies have examined the goose species and their relevant genes; some of the genes, including, CD8α [6], CD4 [4], interleukin(IL)-17A [7], IL-6 [8], Toll like receptor (TLR) 5 [9], MHCI/II [5,10], interferon(IFN)-γ [11], IFN-α [12] and IL-2 [13] were cloned. Despite these studies, many genes related to the goose immune system remain unknown.

To date, the goose transcriptome profiling studies on the identifying the genes responsible for follicle development and reproductive biology in the laying and broodiness [2,14], and comprehensive analyzing the transcriptome of geese to understand the geese development and metabolism [15] has been reported. These research studies were quite different from identifying genes related to the goose immune system. To study the immune system, we are the first to present here the *de novo* transcriptome of Chinese goose (*Anser cygnoides*) peripheral blood lymphocytes (PBLs) using Illumina-Solexa sequencing technology, which is a powerful tool for transcriptome analysis [16]. All of the obtained transcriptome and unigenes were annotated largely from the duck and the chicken, because these two species are closely related to the goose. This study produced the first characterized transcriptome of this waterfowl and provided a genomic picture related to previously undiscovered immune genes. Through the functional annotation of the assembled sequences and identification of the sequenced unigenes, our study identified important novel immune genes related to antigen processing and presentation, toll like receptor signaling pathways, complement cascades, natural killer cell-mediated cytotoxicity response, and inflammatory response by chemokines and cytokine-mediated signaling pathways in the goose.

## Materials and Methods

### Ethics Statement

This study was conducted according to the management regulations of experimental animals in Beijing and was approved by the Animal Care Committee of China Agricultural University, Beijing, People's Republic of China.

### Geese Rearing and Blood Sample Preparation

The five Chinese geese used in this study were raised in the animal isolator house, college of veterinary medicine, China agricultural university, Beijing. These birds were kept under same environmental conditions and provide *ad libitum* water and locally available commercial feed. The blood samples from geese were collected by sterilizing the wing and femoral vein with the surgical cotton containing 70% alcohol. From each bird, 5–10 ml blood was taken and properly mixed with equal volume of EDTA as an anticoagulant, containing tube (1:1) (TBDscience, China).

### Peripheral Blood Lymphocytes Separation and RNA Extraction

For the separation of PBLs, we used a sterile pipette to take 5 ml blood, and added to an equal volume of PBS and mixed well. Then slowly added the blood/PBS mixture on the top of the 10 ml Ficoll-hypaque solution containing tube and centrifuged at 2000 rpm for 20 min. After centrifugation, the tube contained 4 layers (first layer: plasma layer, second layer: white lymphocyte layer, third layer: transparent Ficoll-hypaque layer and fourth layer: erythrocytes). At this point, we removed the upper layer that contains the plasma and most platelets, and the second layer containing lymphocytes was carefully aspirated with a sterile pipette into a new centrifuge tube, which contained 10 ml of washing solution. The tube was thoroughly mixed and centrifuged at 2000 rpm for 20 min. Then removed supernatant, re-suspend cells in washing buffer, and the washing step was repeated twice to obtain the lymphocytes. The total RNA was extracted from the collected Chinese goose lymphocytes using TRIzol (Invitrogen, USA) according to the manufacturer’s protocol. Total RNA was treated with RNase-free DNase I (Promega, USA) for 30 min and then incubated at 37°C to remove residual DNA. The RNA purification was carried out using the RNeasy Mini kit (Qiagen, USA) following the manufacturer’s instructions.

### cDNA Library Construction

Total RNA was prepared to construct the cDNA library and Illumina-Solexa was carried out. In brief, mRNA was isolated and purified from 10 μg of total RNA using oligo(dT) magnetic beads, and short fragments (200–700 bp) were obtained. These short RNA fragments were used as templates for first-strand cDNA synthesis by random hexamer–primers, and then the second-strand cDNA as synthesized by adding buffer, dNTPs, RNAse H and DNA polymerase I. After purification and paired-end (PE) repair, 5’ and 3’ ends of the cDNA fragments were ligated with sequencing adapters and were amplified by polymerase chain reaction (PCR) to generate the templates. The cDNA templates were further enriched by PCR amplification to generate the cDNA library. The cDNA library was sequenced by an Illumina HiSeq 2000 sequencing platform and the raw reads were generated using the Solexa pipeline according to the manufacturer’s instructions.

### *De Novo* Transcriptome Assembly

The raw reads were cleaned by removing adapter sequences, non-coding RNA (such as rRNA, tRNA and miRNA) and low-quality sequences (reads with uncertain bases ‘N’). To insure the quality control of raw read data, we used two steps; the first was the sliding window method to remove low quality segments (Threshold quality 20, window size 5 bp, and threshold length 35 bp), and the second was the removal of reads that contained N as a part of the sequence (Threshold length 35bp). *De novo* transcriptome assembly was performed by the Trinity program [17] (Version r2013/08/14), and the longest transcription sequences were taken and defined as unigenes. To measure RPKM (reads per kilobase of exon model per million mapped reads), the number of sequenced reads that aligned to a gene must be normalized to remove the biases in the aligned sequences [18]. The RPKM was calculated for all assembled unigenes in every sample by single-end mapping using software bowtie2 (version 2.1.0). The unique feature of this tool is that it does not rely on the existence of a reference genome and therefore it is mostly useful for quantification with *de novo* transcriptome assemblies [19]. All unigenes were arranged in descending order from the first unigene. When the assembled length covered half of the total length of all unigenes, the length of the current unigenes was considered to be N50. And when the assembled length covers 90% of the total length, the length of the current unigene was considered to be N90. The sequence database generated in this study is available at the National Center for Biotechnology Information (NCBI) database Short Read Archive under the accession number SRX399106.

### Annotation and Classification of the *De Novo* Transcriptome

All unigenes were searched for homologous genes using BLAST and annotation against the NCBI Nr database (non-redundant, http://www.ncbi.nlm.nih.gov/), using an E-value cut-off of 10^−5^. Unigene sequences were also aligned by BLASTx to various protein databases in the following order: Swiss-Prot and TrEMBL (http://www.ebi.ac.uk/uniprot/), Gene Ontology(GO) (http://www.geneontology.org/), Conserved Domain Database (CDD) (http://www.ncbi.nlm.nih.gov/cdd/), Pfam database (http://pfam.janelia.org/), eukaryotic Orthologous Groups(KOGs) (ftp://ftp.ncbi.nih.gov/pub/COG/KOG/), and Kyoto Encyclopedia of Genes and Genomes (KEGG) (http://www.genome.jp/kegg/). The unigenes were sorted to recover proteins with the most similarity to the given unigenes with putative functional annotations. When the aligned results were different from database sequences, then most privileged results of Nr were selected, followed by the Swiss-port, TrEMBL, CDD, PFAM, GO, KOG and KEGG databases. GO terms at the 2^nd^ level were used to perform the GO annotation of the unigenes under the biological, molecular functions and cellular components. The unigene sequences were also aligned to the KOG database to predict and classify possible functions, and the pathway assignments were performed according to the KEGG pathway database [20].

### Identification and Annotation of the Goose Immunity Relevant Genes

The identification of the most important immunity relevant genes was assembled mainly according to search in our BLAST annotation results to the NCBI databases. A set of keywords representative of immune genes was used to predict immune-related genes based on the annotation results. Similarly, to find the most genes belonging to functions of the immune system, the GO term and KEGG pathway information were also used to identify the most important genes. The immune genes were detected not only as described by [21], but also according to the GO categories “response to stimulus” and “immune system process”, and KEGG pathways “immune system” and “immune diseases,” which had a direct relationship with the immunity genes. Then the finding of the presence and absence of immune relevant genes of goose in comparison with the duck, chicken, turkey and zebra finch were individually search by gene name and identifying regions of the goose immune related genes that were conserved in other species using BLAST annotation results of the NCBI database.

### STRING Analysis

To find out the functional relationship among immune relevant genes, 125 transcripts were imported by selecting *Gallus gallus* as a model organism to STRING 9.1 http://string-db.org/ (a database of known and predicted protein interactions), which responds by displaying a network of nodes (proteins) connected by colored edges representing functional relationships. Interactions of these genes were identified, based on the evidence indicated in the edge map. The STRING database assembles data from genomic context, high-throughput experiments, conserved co-expression and data mining to integrate data from these sources into groups with direct physical and indirect functional associations. Complete knowledge of all direct and indirect interactions between proteins represents an important milestone towards a comprehensive description of cellular mechanisms and functions [22].

### PCR Reaction

PCR was performed to confirm the expression of the recognized immune-related genes. Ten genes, BAFF, C1qA, C1qB, C1qC, SOCS1, SOCS3, TLR3, IL1RL1, C8G and CD74 were utilized to confirm the sequencing data. Genes were selected based on their functions in innate, adaptive immune system and signaling pathways. Primer sequences were designed according to sequences from our transcriptome data. Information of individual primer sequences of the selected 10 genes are listed in S1 Table. For PCR, a TAKARA LA Taq and Primer STAR HS DNA Polymerase kits (Takara Biotechnology (*Dalian*) Co.Ltd) were used according to the manufacturers’ instructions. All reactions were run in triplicate.

### Data Analysis

Amino acid sequences alignment of 10 immune relevant genes (BAFF, C1qA, C1qB, C1qC, SOCS1, SOCS3, TLR3, IL1RL1, C8G and CD74) was analyzed by the ClustalW2.0 program (http://www.ebi.ac.uk/Tools/msa/clustalw2/) and phylogenetic trees of C1qs and SOCSs constructed by the neighbor-joining method with Mega 5.1 software [23].

## Results

### Illumina-Solexa Sequencing and *De Novo* Assembly

**Fig 1:**
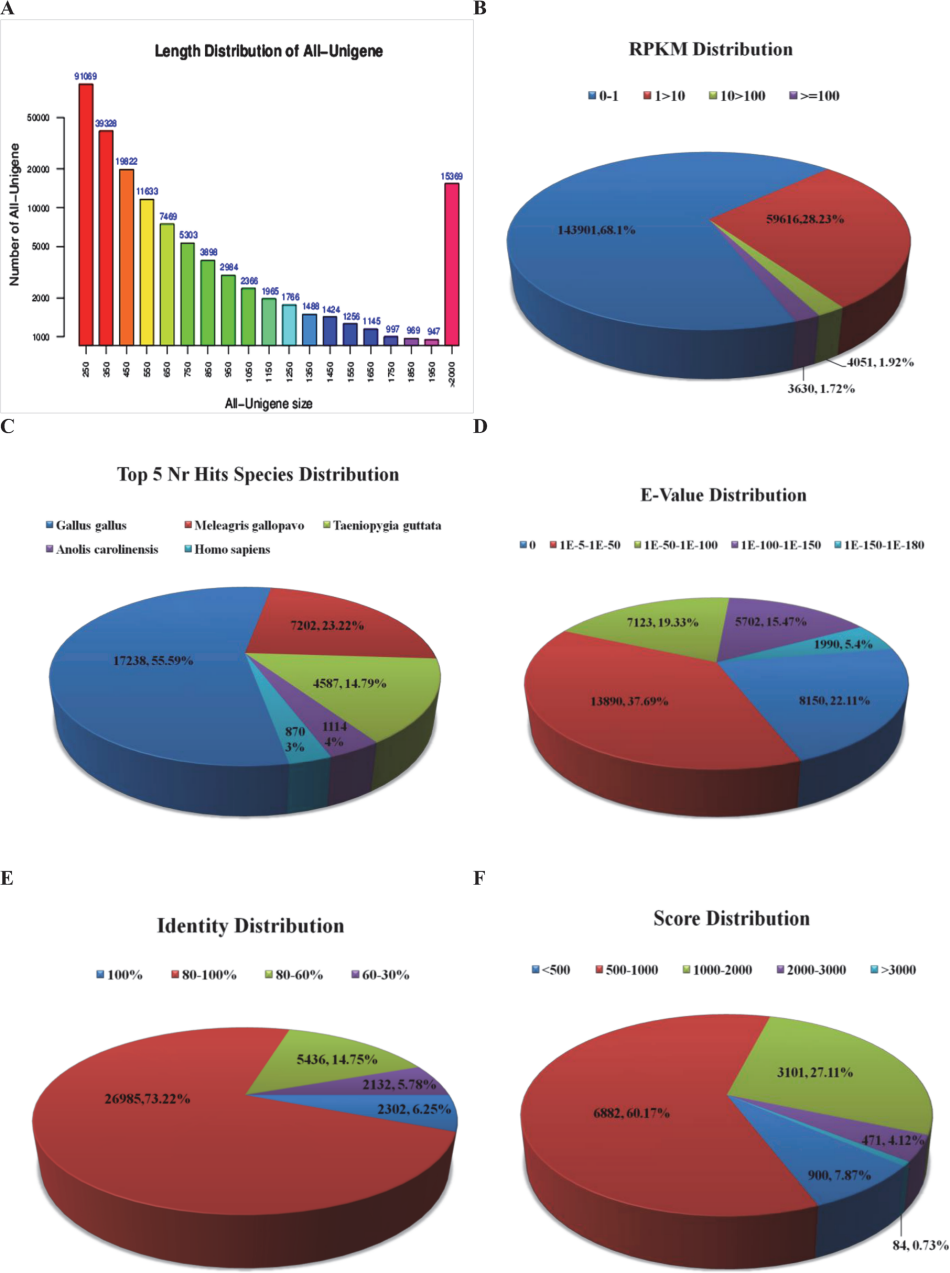
*De novo* transcriptome assembly of goose peripheral blood lymphocytes statistical analysis. **A.** Length distribution of the assembled all unigenes in the sequenced cDNA library, **B.** Distribution of the unigenes in RPKM values, and Characteristics of homology search of assembled unigenes against the Nr database; **(C)** Top 5 species distribution, (**D)** E-value distribution, (**E)** Identity distribution, **(F)** Score distribution.

*De novo* transcriptome sequence data were obtained using Illumina-Solexa deep sequencing to understand the genetic structural design of the goose lymphocyte transcriptome. A cDNA library of goose peripheral blood lymphocytes was sequenced using Illumina-Solexa sequencing technology. Through Solexa RNA paired end sequencing, we generated 91.93 million raw reads with 9.19 Giga base pairs (Gbp) as listed in Table 1. After removing adaptor sequences, ambiguous nucleotides and low-quality sequences, 69.36 million clean reads with a length of 93.65 bp remained. The GC content and average length was 47% and 93.65 bp, respectively. Assembly of all of the clean reads resulted in 211,198 unigenes that ranged from 201 bp to 18,992 bp with the average length of 687 bp and a total length size of 69.5 Mb. Next, all unigenes were sorted in descending order to find the N50 length of 1298 bp and the N90 length of 260 bp. All assembled 211,198 unigene transcripts were more than 200 bp in the length, which indicated that the unigenes were worthy and assured most of the transcriptome sequences. The length distribution of the assembled unigenes in the sequenced cDNA library was shown in Fig. 1A, with the highest number of unigenes (91,069) and the lowest number of unigenes (947) collected under 250 bp and 1950 bp length sizes, respectively.

**Table 1:** Summary of the cDNA library of goose peripheral blood lymphocytes.

Category	Before trimmingRaw Data	After trimmingClean data	After assemblyUnigenes
**Total reads**	91.93 × 10^6^	69.36 × 10^6^	
**Total bases (Gb)**	9.19	6.50	
**Average length (bp)**		93.65	
**Average Ratio**		75.45	
**GC content (%)**		47	
**All (> = 200bp)**			211,198
**> = 500bp**			61,112
**> = 1000bp**			29,716
**N50 (bp)**			1,298
**N90 (bp)**			260
**Max Length (bp)**			18,992
**Min Length (bp)**			201
**Average Length (bp)**			687.87
**Total Length (Mb)**			69.5

The number of clean reads to each annotated unigene was calculated and then normalized by RPKM and bowtie2 software. Out of the 211,198 unigenes, 143,901 (approximately 68.1%) had a RPKM value less than one, 59,616 (28.2%) had a RPKM value between 1 and 10, 4051 (1.9%) had a RPKM value between 10 and 100, and 3630 (1.7%) had a RPKM value of greater than 100 (Fig. 1B).

### BLAST Analysis

The quality control reads data were randomly selected and aligned with the NCBI nucleotide database. For each query, we selected the best one among all of the aligned sequences with an E-value of 10^−10^ and that covered more than 80% similarity. All unigene sequences were aligned against the public database using the BLAST comparison to known sequence databases and functional annotation with similarity >30% and an E-value cut of 10^−5^. The alignment results of the public databases (Nr, Swissport, TrEMBL, Pfam, CDD, KOG and KEGG) were shown in Table 2. The aligned sequences were matched with the Nr 17.44%, TrEMBL 17.70% and SWISSPORT 15.14% databases, respectively. The 36,854 (17.44%) unigene BLAST hits in the Nr database were studied as the Nr database had the maximum annotated unigenes. However, the alignments with Nr database were in the top five hits species (Fig. 1C), and most of the aligned sequences were matched to the *Gallus gallus* 17,238 (55.59%). The E-value distribution showed that 13,890 (37.7%) unigenes were significantly homologous with a significance of 1E-5 to 1E-50, and 7123 unigenes were in the range from 1E-50 to 1E-100 (Fig. 1D). Similarly, the identity distribution of unigenes revealed that 26,985 (73.2%) unigenes were highly matched to 80–100% identity and 5436 (14.7%) unigenes were between 60–80% matched (Fig. 1E). In addition, the score distribution showed that 900 (7.8%) unigenes had scores less than 500, 84 (0.7%) unigenes had scored more than 3,000, and 6882 unigenes (60.1%), which was the greatest number, had a score between 500 and 1000 (Fig. 1F).

**Table 2:** List of annotations against public databases.

Annotation database	Number of annotated unigenes	Percentage of annotated unigenes
**TOTAL**	211198	
**NR**	36854	17.44%
**SWISS-PROT**	31978	15.14%
**TREMBL**	37394	17.70%
**CDD**	17981	8.51%
**PFAM**	32498	15.38%
**KOG**	15334	7.26%
**KEGG**	39,585	18.74%

### Functional Annotation and Pathway Classification

**Fig 2:**
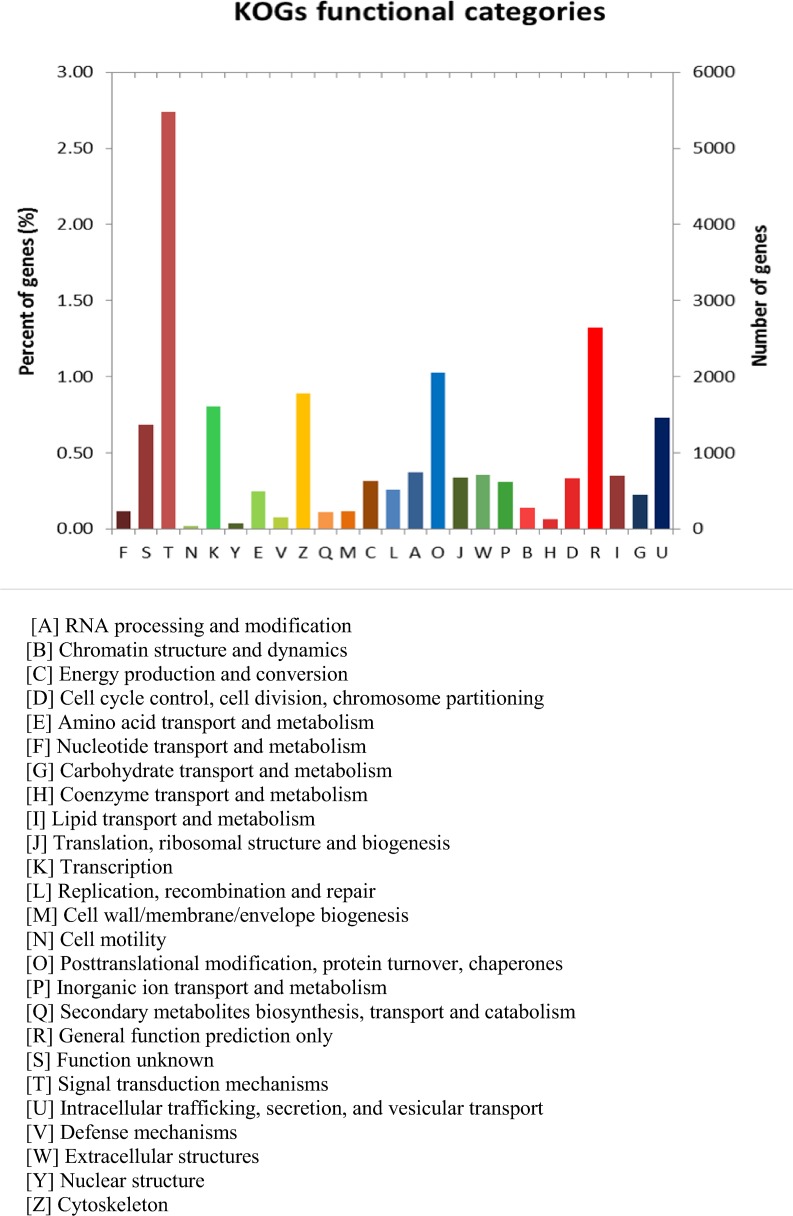
Histogram presentation of eukaryotic cluster of orthologous groups (KOG) classification. A total of 15,334 sequences were annotated into 25 KOG categories.

The rapid assembly of genome sequences is a major challenge to researchers attempting to extract the maximum functional and evolutionary information from the genome data. The NCBI eukaryotic orthologous groups (KOGs) include sequences from 7 eukaryotic genomes [24]. To further evaluate the transcriptome library, the accuracy of our annotation sequences in the KOG functional classification was examined. All unigenes were aligned to the KOG database for prediction and classification into different functional categories. There were 15,334 unigenes annotated into 25 KOG categories (Fig. 2). The largest group was signal transduction mechanism at 5787 (2.74%) followed by the general function prediction only at 2792 (1.32%), posttranslational modification, protein turnover, chaperones at 2168 (1.02%), and cytoskeleton at 1876 (0.88%). Nuclear structure 75 (0.03%) and cell motility 45 (0.02%) were the KOG categories with the least represented unigenes.

**Fig 3:**
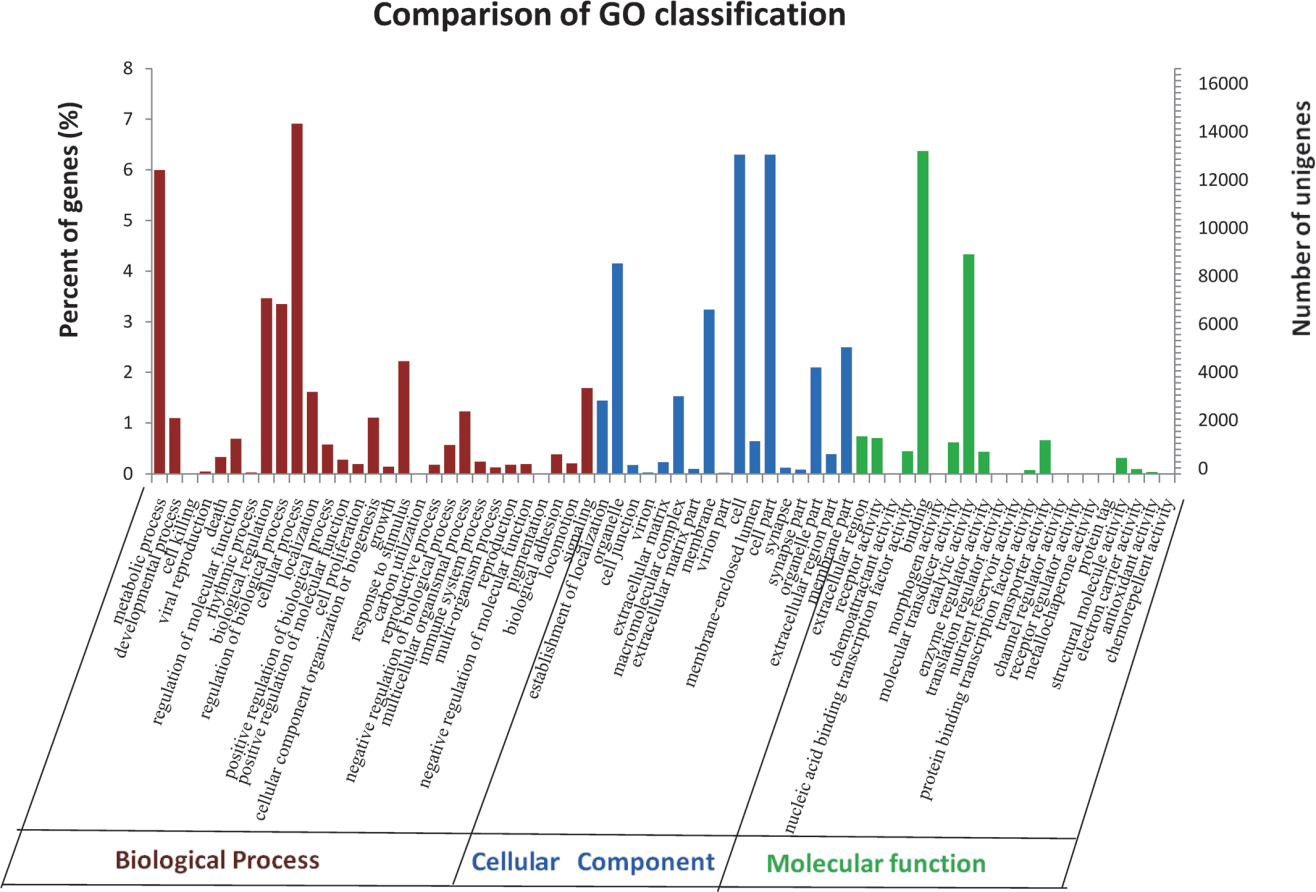
Histogram of Gene Ontology (GO) Classification. The terms were summarized in three main categories (biological process, cellular component and molecular function) and 67 sub-categories. Right y-axis, percentage of unigenes; Left y-axis, number of unigenes.

Gene Ontology (GO) is an international standardized gene functional classification system that uses strict definitions to comprehensively describe the properties of genes and their products in any organism [25]. A total of 163,161 unigenes were assigned in GO annotations and were divided into three categories and 67 functional categories. The biological process category contained 72,844 unigenes, followed by the 60,500 in the cellular component category and 29,817 in molecular function category (Fig. 3). In the biological process category, ‘cellular process’ at 14,596 (8.95%), ‘metabolic process’ at 12,660 (7.75%) and biological regulation at 7317 (4.48%) were the most highly represented. In addition, unigenes were categorized into other 27 important biological processes, including ‘regulation biological process’ at 7075 (4.33%) unigenes, ‘response to stimulus’ at 4686 (2.8%) unigenes, ‘signaling’ at 3570 (2.2%) unigenes and ‘immune system process’ at 512 (0.3%) unigenes, which were mainly involved in resistance or the defense system in the geese. However, 17 GO functional groups were assigned to the cellular component category with ‘cell’ and ‘cell part’ with the same number of unigenes at 13,309 (8.15%), followed by ‘organelle’ at 8771 (5.37%) and ‘membrane’ at 6,841 (4.19%) as the most represented. Likewise, 20 GO functional groups were assigned into the molecular function category with ‘binding’ at 13,457 (8.2%) and ‘catalytic activity’ at 9,144 (5.60%) as the most highly represented.

**Fig 4:**
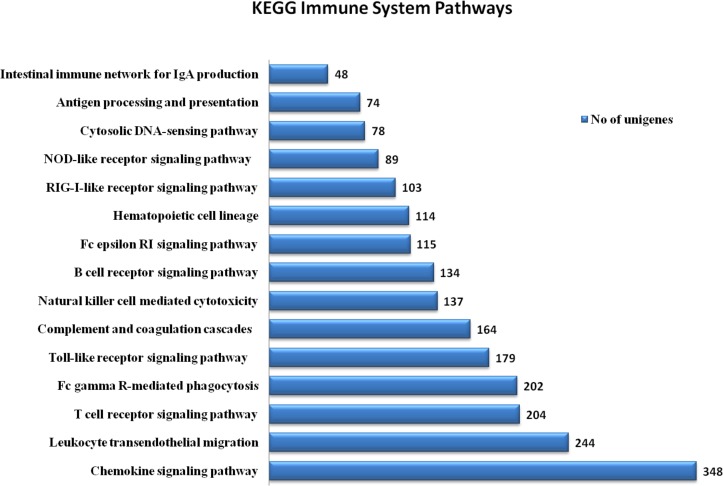
Distribution of unigenes involved in the immune system pathways. A total of 2757 unigenes were involved in the immune system.

To evaluate the biological system of the geese, we aligned the annotated sequences to the corresponding KEGG pathways and analyzed the relationship between unigene and pathways to further understand the biological functions and gene interactions [20]. Out of 211,198 unigenes, 39,585 (18.74% of total unigenes) KEGG pathways and annotated unigenes were categorized into six biological functional groups (Table 3). A total of 39,585 unigenes had significant matches in the database and were assigned to 308 KEGG pathways. Some of unigenes were mapped to more than one pathway. Among unigenes mapped into pathways, the highest numbers of unigenes were involved in the human disease category (approximately 28.45%). Other unigenes were assigned into pathways of organismal processes (24.77%), metabolism (14.54%), cellular processes (13.35%), environmental information processing (11.7%) and genetic information processing (7.16%). The lymphocytes have pivotal functions in cell-mediate immunity: the innate (by NK cells) and adaptive (by T-cells) immune systems, as well as the antibody derived humoral (by B cells) immune response, are the main functions of lymphocyte [25]. Our present analysis show that large numbers of the unigenes lye in the immune relevant pathways. Out of the six groups listed above, human diseases and organismal systems contained the most unigenes. Within organismal systems, it is noteworthy that the immune systems group, comprised 2757 (6.96%) out of 39,585 unigenes were involved in 17 KEGG pathways (Table 3). The immune system pathways were further categorized into 15 subcategories as shown in Fig. 4. Among subcategories, the chemokine signaling pathways comprised of highest number of unigenes at 348 followed by leukocyte transendothelial migration at 244 unigenes, T-cell receptor signaling pathway at 204 unigenes, Fc gamma R-mediate phagocytosis at 202 unigenes, Toll-like receptor signaling pathway at 179 unigenes, complement and coagulation cascades at 164 unigenes, natural killer cell mediated cytotoxicity at 137 unigenes, B-cell receptor signaling pathway at 134 unigenes, Fc epsilon RI signaling pathway at 115 unigenes, hematopoietic cell lineage at 114 unigenes, RIG-I-like receptor signaling pathway at 103 unigenes, NOD-like receptor signaling pathway at 89 unigenes, cytosolic DNA-sensing pathway at 78 unigenes, antigen processing and presentation at 74 unigenes, and intestinal immune network for IgA production at 48 unigenes. Out of 2757 unigenes, 125 of the most important immunity relevant genes were identified from the goose transcriptome and are summarized in Table 4.

**Table 3:** Classification of KEGG pathway unigenes from the cDNA library of Goose peripheral blood lymphocytes.

KEGG Category	Number of Unigenes	Percentage ofUnigenes	Number of KEGG pathways
**Metabolism**		**14.54%**	
Amino acid metabolism	742		12
Biosynthesis of other secondary metabolites	91		11
Carbohydrate metabolism	1004		14
Energy metabolism	516		9
Glycan biosynthesis and metabolism	767		14
Lipid metabolism	960		15
Metabolism of cofactors and vitamins	345		12
Metabolism of other amino acids	321		9
Metabolism of terpenoids and polyketides	55		8
Nucleotide metabolism	624		2
Xenobiotics biodegradation and metabolism	336		17
**Genetic information processing**		**7.16%**	
Folding, Sorting and Degeneration	936		7
Replication and repair	595		7
Transcription	386		3
Translation	917		5
**Environmental information processing**		**11.7%**	
Membrane transport	155		2
Signal transduction	3145		15
Signaling molecules and interaction	1334		4
**Cellular processes**		**13.35%**	
Cell communication	2205		7
Cell growth and death	1140		7
Cell motility	493		1
Transport and catabolism	1448		6
**Organismal systems**		**24.77%**	
Circulatory system	1032		4
Development	663		3
Digestive system	1372		9
Endocrine system	1277		6
Environmental adaptation	137		4
Excretory system	409		5
Immune system	2757		17
Nervous system	1816		8
Sensory system	345		5
**Human diseases**		**28.45%**	
Cancers	3871		17
Cardiovascular diseases	1325		5
Endocrine and metabolic diseases	217		3
Immune diseases	337		7
Infectious diseases	4449		22
Neurodegenerative diseases	1063		5

**Table 4:** Comparison of immune relevant genes of goose with duck, chicken, turkey and zebra finch.

Unigenes	Gene Symbol	Gene description	Duck	Chicken	Turkey	Zebra finch
**Antigen Processing and presentation**						
**comp58948_c2**	MHCI*	Major histocompatibility complex, class I	Present	Present	Present	Present
**comp56480_c0**	MHCII	Major histocompatibility complex, class II	Present	Present	Present	Present
**comp59549_c0**	β2m	Beta-2-microglobulin	Present	Present	Present	Present
**comp39750_c0**	TAPBP	TAP binding protein (tapasin)	Absent	Present	Absent	Absent
**comp41693_c0**	HSP90A	Heat shock protein 90KDa alpha	Present	Present	Absent	Present
**comp29626_c0**	HSP70*	Heat shock protein 70	Present	Present	Absent	Present
**comp59973_c0**	CTSS	Cathepsin S	Present	Present	Present	Present
**comp30827_c1**	CD8b	T-cell surface glycoprotein CD8 beta	Present	Present	Present	Present
**comp52413_c0**	CD74*	MHC II invariant chain CD74 protein	Present	Present	Present	Present
**comp48887_c0**	RFX5	Regulatory factor X 5 (influence on HLA II expression)	Present	Present	Absent	Present
**Toll-like receptor signaling pathway**						
**comp128330_c0**	TLR2	Toll-like receptor 2	Present	Present	Present	Present
**comp35851_c0**	TLR3*	Toll-like receptor 3	Present	Present	Absent	Present
**comp53805_c0**	TLR4*	Toll-like receptor 4	Present	Present	Present	Present
**comp93690_c0**	TLR5*	Toll-like receptor 5	Present	Present	Present	Present
**comp74903_c0**	TLR7*	Toll-like receptor 7	Present	Present	Present	Present
**comp30216_c0**	TLR13	Toll-like receptor 13	Absent	Present	Absent	Absent
**comp140074_c0**	TLR15*	Toll-like receptor 15	Present	Present	Absent	Absent
**comp21608_c0**	TLR18	Toll-like receptor 18	Absent	Absent	Absent	Absent
**comp49398_c0**	TICAM1	Toll-like receptor adapter molecule 1	Present	Absent	Absent	Absent
**comp43055_c0**	FADD	FAS-associated death domain protein	Present	Present	Present	Present
**comp56547_c0**	LY75	Lymphocyte antigen 75	Present	Present	Present	Present
**comp53505_c0**	LY96	Lymphocyte antigen 96	Present	Present	Absent	Present
**comp59404_c1**	MYD88	Myeloid differentiation primary response protein MyD88	Present	Present	Present	Present
**Chemokine signaling pathway**						
**comp40528_c0**	CCL4	C-C motif chemokine 4	Present	Present	Present	Present
**comp32248_c0**	CCL14	C-C motif chemokine 14	Present	Absent	Absent	Absent
**comp60229_c0**	CCL19	C-C motif chemokine 19	Present	Present	Present	Absent
**comp48236_c0**	CCL20	C-C motif chemokine 20	Present	Present	Present	Present
**comp103248_c0**	CCL26	C-C motif chemokine 26	Absent	Absent	Present	Absent
**comp53780_c0**	CCR7	C-C chemokine receptor 7	Present	Present	Absent	Present
**comp150987_c0**	CCR8	C-C chemokine receptor 8	Present	Present	Present	Present
**comp194697_c0**	CCR9	C-C chemokine receptor 9	Present	Present	Absent	Present
**comp59998_c0**	CXCR4	CXC chemokine receptor 4	Present	Present	Present	Present
**comp52384_c0**	CXCR7	CXC chemokine receptor 7	Present	Present	Absent	Present
**comp50320_c0**	CX3CR1	CX3C chemokine receptor 1	Present	Present	Absent	Present
**comp74139_c0**	XCR1	XC chemokine receptor 1	Present	Present	Absent	Present
**comp92058_c0**	NOX1	NADPH oxidase	Present	Present	Present	Present
**Cytokine-cytokine receptor interaction**						
**comp34116_c0**	IL1B*	Interleukin 1 beta	Absent	Present	Present	Present
**comp17611_c0**	IL6*	Interleukin 6	Present	Present	Present	Present
**comp16190_c0**	IL8*	Interleukin 8	Absent	Present	Present	Present
**comp160975_c0**	IL16	Interleukin 16	Present	Present	Present	Present
**comp181708_c0**	IL17*	Interleukin 17	Present	Present	Present	Present
**comp27858_c0**	IL18*	Interleukin 18	Present	Present	Present	Present
**comp41212_c0**	IL1R1	Interleukin 1 receptor type 1	Present	Present	Present	Present
**comp47155_c0**	IL1R2	Interleukin 1 receptor type 2	Present	Present	Present	Present
**comp39273_c0**	IL2RA*	Interleukin 2 receptor alpha	Absent	Present	Absent	Absent
**comp50517_c0**	IL2RB	Interleukin 2 receptor beta	Present	Present	Absent	Present
**comp47144_c0**	IL2RG	Interleukin 2 receptor gamma	Present	Present	Absent	Present
**comp40605_c0**	IL4R	Interleukin 4 receptor	Present	Present	Present	Present
**comp129591_c0**	IL5RA	Interleukin 5 receptor alpha	Present	Present	Absent	Present
**comp195264_c0**	IL9R	Interleukin-9 receptor	Absent	Present	Absent	Absent
**comp40832_c0**	IL10RA	Interleukin 10 receptor alpha	Present	Present	Present	Present
**comp39646_c1**	IL10R2	Interleukin 10 receptor 2	Present	Present	Present	Present
**comp52039_c0**	IL11RA	Interleukin 11 receptor alpha	Present	Present	Present	Present
**comp10084_c0**	IL12RB2	Interleukin 12 receptor beta-2	Present	Present	Present	Absent
**comp56559_c0**	IL13RA1	Interleukin 13 receptor alpha-1	Present	Present	Absent	Absent
**comp56492_c1**	IL13RA2	Interleukin 13 receptor alpha-2	Present	Present	Present	Present
**comp39945_c1**	IL15RA	Interleukin 15 receptor alpha	Absent	Present	Absent	Absent
**comp53179_c0**	IL20RA	Interleukin 20 receptor alpha	Present	Present	Present	Present
**comp47289_c0**	IL21R	Interleukin 21 receptor	Present	Present	Present	Present
**comp39893_c0**	IL23R	Interleukin 23 receptor	Present	Present	Absent	Absent
**comp14961_c0**	IL27B	Interleukin 27 subunit beta	Absent	Absent	Absent	Absent
**comp72820_c0**	IL28RA	Interleukin 28 receptor subunit alpha	Absent	Present	Absent	Absent
**comp78264_c0**	IL31RA	Interleukin 31 receptor subunit alpha	Present	Present	Absent	Absent
**comp57283_c0**	IRAK2	Interleukin-1 receptor-associated kinase 2	Present	Present	Present	Present
**comp77312_c0**	BMP2	Bone morphogenetic protein 2	Present	Present	Present	Present
**comp55696_c0**	TGFBR2	TGF-beta receptor-2	Present	Present	Present	Present
**Transcription factors for immune response**						
**comp42892_c0**	IFNGR1	Interferon gamma receptor 1	Present	Present	Present	Present
**comp46691_c1**	IRF1	Interferon regulatory factor 1	Present	Present	Present	Present
**comp49737_c0**	IRF2	Interferon regulatory factor 2	Present	Present	Present	Present
**comp53383_c0**	IRF3	Interferon regulatory factor 3	Absent	Absent	Present	Present
**comp56408_c0**	IRF4	Interferon regulatory factor 4	Present	Present	Present	Present
**comp53680_c1**	IRF5	Interferon regulatory factor 5	Present	Present	Present	Present
**comp41288_c0**	IRF6	Interferon regulatory factor 6	Present	Present	Present	Present
**comp53383_c0**	IRF7	Interferon regulatory factor 7	Absent	Present	Absent	Absent
**comp56307_c0**	IRF8	Interferon regulatory factor 8	Present	Present	Present	Present
**comp112074_c0**	IRF9	Interferon regulatory factor 9	Absent	Absent	Absent	Absent
**comp38916_c0**	IRF10	Interferon regulatory factor 10	Absent	Present	Absent	Absent
**comp50302_c0**	MX	Interferon-induced GTP-bindingprotein Mx	Present	Present	Present	Present
**comp53021_c1**	BLNK	B-cell linker protein	Present	Present	Absent	Present
**comp57373_c0**	NOD1	Nucleotide-binding oligomerization domain-containing protein 1	Present	Present	Present	Present
**comp45455_c0**	OAS	2'-5'-oligoadenylate synthetase	Present	Present	Absent	Absent
**Complement and coagulation cascades**						
**comp26818_c0**	C2	Complement C2	Absent	Present	Absent	Absent
**comp58468_c0**	C4	Complement C4	Present	Present	Present	Present
**comp54874_c0**	C4–1*	Complement C4–1	Absent	Absent	Absent	Absent
**comp57162_c0**	C5	Complement C5	Present	Present	Present	Present
**comp59644_c0**	C3	Complement component 3	Present	Present	Present	Present
**comp51375_c0**	C4BPA	Complement component 4 binding protein, alpha	Absent	Present	Absent	Absent
**comp16085_c0**	C7	Complement component C7	Absent	Absent	Absent	Absent
**comp54175_c0**	C8B	Complement component 8 beta	Absent	Absent	Absent	Absent
**comp44323_c0**	C8G	Complement component C8 gamma chain	Present	Present	Absent	Present
**comp49143_c0**	C1R	Complement component 1, r subcomponent	Present	Present	Present	Present
**comp29708_c0**	C1S	Complement component 1, s subcomponent	Present	Present	Present	Present
**comp44414_c0**	C1qA	Complement C1q subcomponent subunit A	Present	Present	Present	Present
**comp44360_c0**	C1qB	Complement C1q subcomponent subunit B	Present	Present	Present	Present
**comp53045_c0**	C1qC	Complement C1q subcomponent subunit C	Present	Present	Present	Present
**comp16573_c0**	CFI	Complement factor I	Present	Present	Present	Present
**comp59485_c0**	MBL	Mannose-binding lectin	Present	Present	Absent	Absent
**comp38813_c0**	A2M	alpha-2-macroglobulin	Present	Present	Present	Present
**comp44369_c0**	KNG1	Kininogen-1	Present	Present	Absent	Present
**Natural killer cell mediated cytotoxicity**						
**comp48258_c0**	PRF1	Perforin-1	Absent	Present	Absent	Absent
**comp49302_c0**	NFATC. NFAT	Nuclear factor of activated T-cells. cytoplasmic	Present	Present	Present	Present
**comp58097_c0**	MAP3K14	Mitogen-activated protein kinase 14	Present	Present	Present	Present
**comp62022_c0**	LCK	Lymphocyte-specific protein tyrosine kinase	Absent	Present	Absent	Absent
**comp71765_c0**	PAK1	p21-activated kinase 1	Present	Present	Present	Present
**comp46839_c0**	ZAP70	Zeta-chain (TCR) associated protein kinase	Present	Present	Present	Present
**comp56811_c0**	LCP2	Lymphocyte cytosolic protein 2	Present	Present	Present	Present
**comp70056_c0**	MST1R	Macrophage stimulating 1 receptor	Present	Present	Present	Present
**comp59359_c0**	PTPN1	Protein tyrosine phosphatase, non-receptor type 1	Present	Present	Present	Present
**comp50816_c0**	PTPN11	Protein tyrosine phosphatase, non-receptor type 11	Present	Present	Present	Present
**comp12857_c0**	CTLA4	Cytotoxic T-lymphocyte-associated protein 4	Present	Present	Present	Present
**NF-kappa B signaling pathway**						
**comp36510_c0**	NFKBIB	NF-kappa-B inhibitor beta	Absent	Present	Absent	Present
**comp53597_c0**	NFKBIE	NF-kappa-B inhibitor epsilon	Present	Present	Present	Present
**comp54864_c0**	CARD11	Caspase recruitment domain-containing protein 11	Present	Present	Present	Present
**comp53329_c0**	BTK	Bruton agammaglobulinemia tyrosine kinase	Present	Present	Present	Present
**comp58969_c0**	MALT1	Mucosa-associated lymphoid tissue lymphoma translocation protein 1	Present	Present	Present	Present
**comp56822_c0**	NFKB	Nuclear factor NF-kappa-B	Present	Present	Present	Absent
**comp58131_c0**	TRAF3	TNF receptor-associated factor 3	Present	Present	Present	Present
**comp59064_c3**	BAFF*	B-Cell activating factor	Present	Present	Absent	Absent
**comp48557_c0**	VCAM1	Vascular cell adhesion molecule 1	Present	Present	Absent	Present
**comp55630_c0**	TNFAIP3	Tumor necrosis factor, alpha-induced protein 3	Present	Present	Present	Present
**Jak-STAT signaling pathway**						
**comp15203_c0**	SOCS1	Suppressor of cytokine signaling 1	Present	Present	Present	Present
**comp42000_c0**	SOCS3	Suppressor of cytokine signaling 3	Present	Present	Present	Present
**comp50037_c0**	CBL	E3 ubiquitin-protein ligase CBL	Present	Present	Present	Present
**comp58567_c0**	STAT1	Signal transducer and activator of transcription 1	Present	Present	Present	Present
**comp37694_c0**	STAT3	Signal transducer and activator of transcription 3	Present	Present	Present	Present

Gene symbols with mark * are already identified in goose species.

### Identification of Immunity Relevant Genes

**Fig 5:**
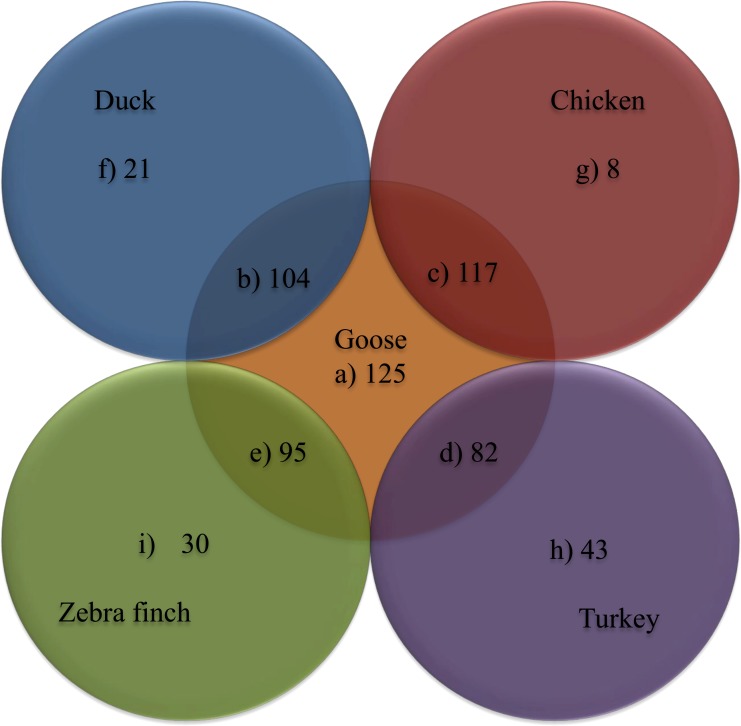
Comparison of goose immune relevant genes with duck, chicken, turkey and zebra finch. The number of immune relevant genes of goose and its comparison among the known sequences of duck, chicken, turkey and zebra finch deposited in the NCBI database. (a) represents the total number of immune relevant genes found in the goose, (b-e) represents the immune relevant genes found in duck, chicken, turkey, and zebra finch, and (f-i) represents the immune relevant genes not found in the duck, chicken, turkey, and zebra finch.

Lymphocytes play an important role in the immune response and each type playing different roles: B lymphocytes are more associated with humoral immunity, while T-cells are the main players in cell-mediated immunity [26]. In this study, we were interested in identifying immune-related genes in the transcriptome of the goose PBLs. According to the literature and our sequence analyses, 125 immune-related genes were identified (Table 4). We identified the most important genes that are relevant to immunity in different KEGG pathways. The antigen processing and presentation category contained 10 unigenes, including MHCI, MHCII, TAPBP. The toll-like receptor signaling pathway category contained 13 unigenes, including TLR-2, −3, −4, −5, −7, −13, −15, −18, FADD, LY96, MyD88. The chemokine signaling pathway category contained 14 unigenes, including CCLs, CCRs, CXCRs, XCR1. The cytokine-cytokine receptor interaction category contained 30 unigenes, including ILs, ILRs, IRAK, BMP2, TGFBR. The transcription factors for immune response category contained 15 unigenes, including IFRs, MX, BLNK, NOD1. The complement and coagulation cascades category contained 18 unigenes, including C2, C3, C5, C8, C1S, C1R, C1qA-C, MBL, A2M. The natural killer cell mediates cytotoxicity category contained 11 unigenes unigenes, including PRF1, NFAT, MAP3K14, LCK, PAK1, ZAP70, LCP2, MST1R, PTPN1, CTLA4 The NF-kappa B signaling pathway category contained 10 unigenes, including NFKBI, CARD11, BTK, MALT1, TRAF3, BAFF. The Jak-STAT signaling pathway category contained 5 unigenes, including SOCS1, SOCS3, CBL, STAT1, STAT3 (Table 4). Of the 125 unigenes, only 16 (approximately 13%) unigenes from the known goose species (*anser anser* 9, and *anser cygnoides* 7) were identified using an NCBI database blast search. We also searched by gene name and by identifying regions of the goose immune related genes that were conserved in other species using BLAST. We found that the majority of the immune genes could be identified in duck, chicken, turkey and zebra finch, but some genes appeared to be unique to geese (Shows in Fig. 5 and Table 4). In conclusion, 109 unigenes (approximately 87%) were new genes and were not previously identified in the goose.

In order to confirm these immune-related genes in goose and analysis their relationships with those of other species, we cloned some genes from innate, adaptive immune system and signaling pathways (S1 Fig.).

**Fig 6:**
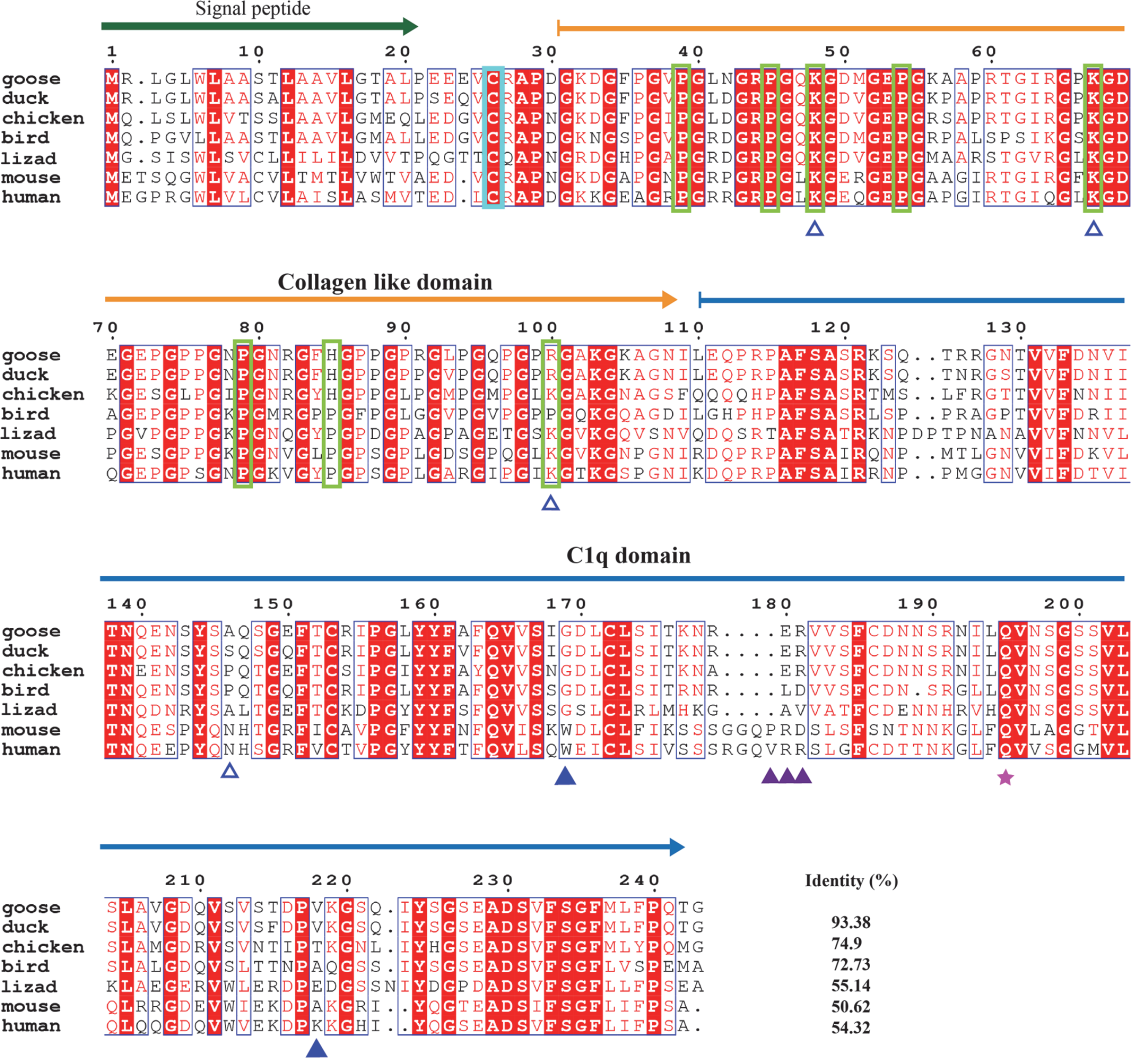
Amino acid alignment of complement C1q subcomponent subunit A (C1qA). Amino acid alignment of C1qA shows, signal peptide, collagen like domain and C1q domain are respectively indicated with a bottle green arrow, orange arrow and blue arrow. Blue Triangle indicates the key residues for CRP interaction. Purple Triangle indicates the key residues for IgG interaction. Magenta pentacle indicates the sites for calcium binding. Residues in the cyan rectangle are in the formation of inter-chain disulfide bond. Residues with the hollow blue triangle are O-linked glycans sites in post-translation. Residues in yellow-green rectangle are hydroxylation sites with GXPG motif. NCBI accession numbers of C1qAs are listed as follows: goose: KP238277; duck: 514725938; bird (zebra finch): 449487169; chicken: 118101238; lizard: XP_003230642.1; mouse: 408359988; human: 399138.

**Fig 7:**
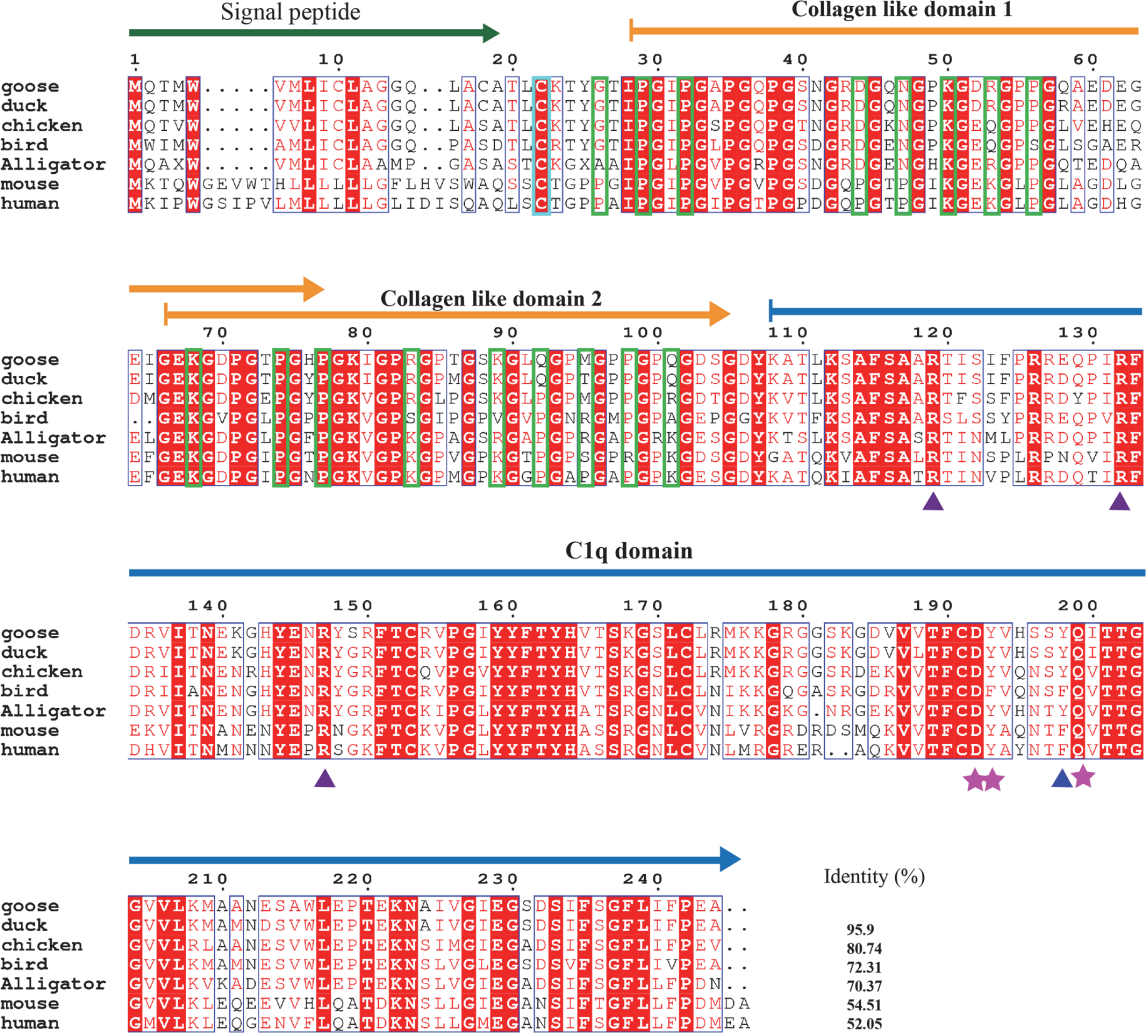
Amino acid alignment of complement C1q subcomponent subunit B (C1qB). C1qA amino acid sequence alignments shows the signal peptide, collagen like domain and C1q domain are respectively indicated with bottle green arrow, orange arrow and blue arrow. Blue Triangle indicates the key residues for CRP interaction. Purple Triangle indicates the key residues for IgG interaction. Magenta pentacle indicates the sites for calcium binding. Residues in the cyan rectangle are in the formation of inter-chain disulfide bond. Residues in yellow-green rectangle are hydroxylation sites. NCBI accession numbers of C1qBs are listed as follows: goose: KP238278; duck: 514725930; chicken: 118101234; bird (zebra finch): 449487165; alligator: 557280613; mouse: 6753220; human: 87298828;

**Fig 8:**
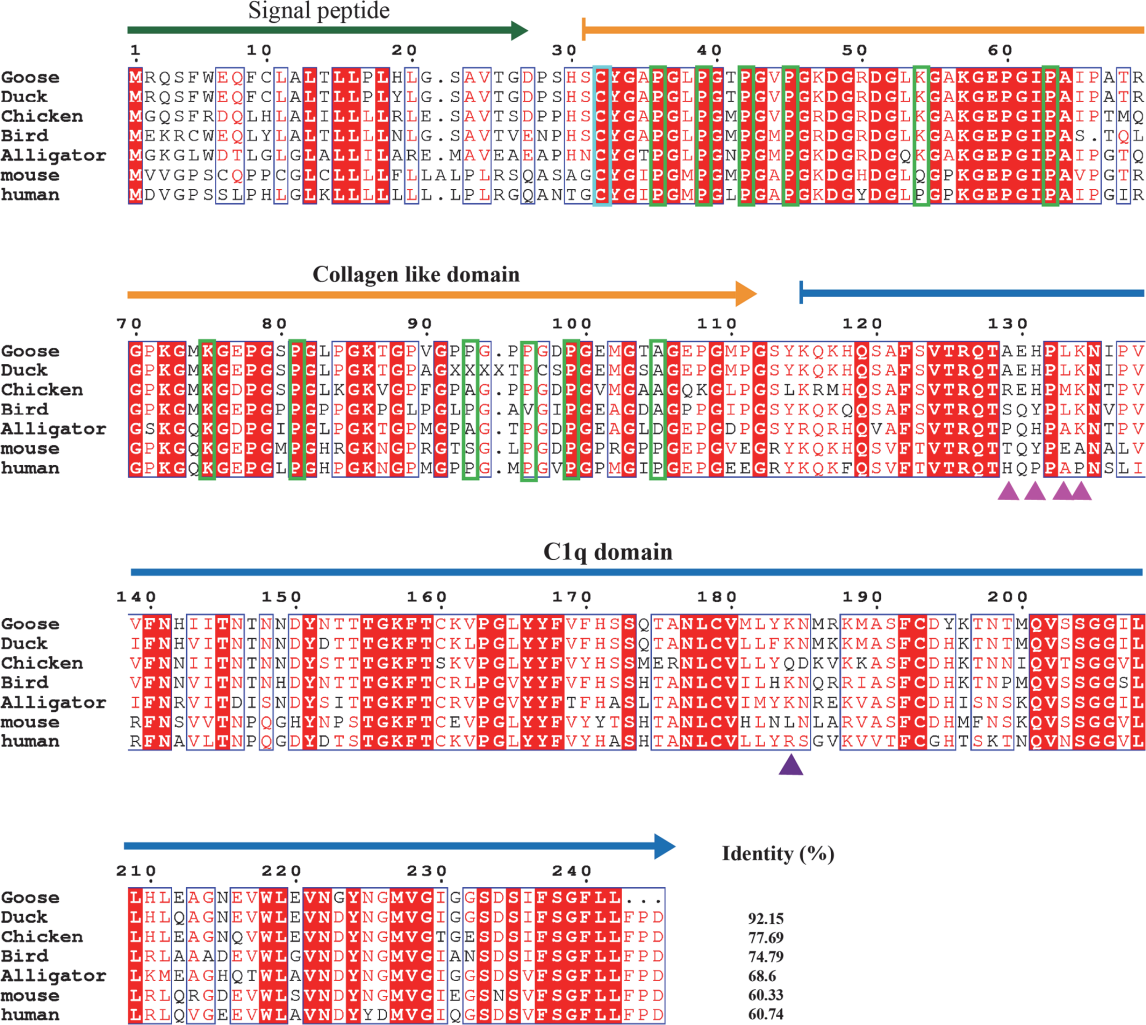
Amino acid alignment of complement C1q subcomponent subunit C (C1qC). Amino acid alignment of C1qC shows the signal peptide, collagen like domain and C1q domain are respectively indicated with a bottle green arrow, orange arrow and blue arrow. Blue Triangle indicates the key residues for CRP interaction. Purple triangle indicates the key residues for IgG interaction. Magenta triangle indicates the sites for HTLVI gp21 peptide binding. Residues in the cyan rectangle are in the formation of inter-chain disulfide bond. Residues in yellow-green rectangle are hydroxylation sites. NCBI accession numbers of C1qCs are listed as follows: goose: KP238279; duck: 514725934; chicken: 50759463; bird (zebra finch):449487244; alligator: 557280504; mouse: 113680120; human: 56786155;

The complement system, which helps antibodies and phagocytic cells eliminate infectious microbes and cellular debris, is one of the key components of the innate immune system. C1q, the ligand-binding unit of the C1 complex of complement, is the first subcomponent of the classical pathway and is a major link between innate and adaptive immunity. Many studies have examined mammalian C1q, but least information is known about avian C1qs, especially C1q in the goose. Here, the A, B and C chains of the goose C1q have been cloned (S1 Fig.). The mature peptides of the C1qA, B, C chains are 243, 244 and 242 amino acids in size, respectively (Figs. 6–8). Goose C1qA, B, and C have similar molecular structures comprised of a signal peptide, one (C1qA and C1qC) or two (C1qB) collagen-like domains and a C1q domain. This structural arrangement is also conserved in other species, such as reptiles and mammals. When the species are compared, the goose C1qA, B and C chains all have the highest identities to duck C1qs (93.38% for C1qA, 95.9% for C1qB and 92.15% for C1qC), followed by chicken and zebra finch (bird) C1qs. In the blood, C1qA, B and C form a heterotrimer that is stabilized by interchain disulfide bonds. The sites for the formation of the disulfide bonds are Cys26 in goose C1qA, Cys22 in goose C1qB and Cys32 in goose C1qC and all these sites are conserved from birds to humans.

Most of the sites for glycosylation, hydroxylysine hydroxylation and hydroxyproline hydroxylation found in the collagen-like regions of human C1qs [27] can also be found in the goose C1qs. The variant sites in the goose C1qA chain are His85, Arg100 and Ala146. The variant sites in the goose C1qB chain are Asp44, Asn47, Arg53, Arg83, Gln92, Met95, and Gln101. In the formation of complement component C1, the collagen-like regions of C1qs are supposed to be recognized by the modular proteases C1r and C1s. A motif shared between C1qA, B and C, Hyp-Gly-Lys-X-Gly-Pro/Tyr/Asn (where Hyp is hydroxyproline), mediates binding to C1r and C1s [28]. Similar regions are also observed in the goose C1q chains, such as Hyp79-Phe84 in C1qA, Hyp77-Pro82 in C1qB and Hyp84-Pro89 in C1qC. Among them, the C1r-C1s binding regions are most conserved in C1qB, followed by C1qC. Most of the variations are found in C1qAs, especially among the avian species.

As a versatile pattern recognition molecule, the heterotrimeric globular domain (gC1q) of C1q is thought to be capable of engaging a broad range of ligands, including aggregated IgG and IgM, C-reactive protein (CRP), human T cell lymphotropic virus-I (HTLV-I) gp21 peptide [29]. Val183, Arg184, Arg185 in human C1qA, Arg126, Arg139 and Arg154 in human C1qB and Arg184 in human C1qC are the key sites for IgG interaction. These sites are strictly conserved in the goose C1qB and in other C1qBs. These sites are not seen in the C1qAs and C1qCs of other species, even in a mammal (mouse). The Lys200 and Trp147 sites of human C1qA that interact with CRP are not found in other species, except for in the mouse. Tyr198 of the human C1qB is relatively well conserved among different species because the same or similar amino acids are found in other C1qBs. In contrast, His129, Pro131, Ala133 and Pro134 of human C1qC, which are important in binding to the HTLVI gp21 peptide, are not observed in other C1qCs. However, the calcium ion binding sites of C1qs are strictly conserved from geese to humans. These sites are Gln195 in goose C1qA, and Asp192, Tyr193 and Gln199 in goose C1qB. The different conservation of binding sites may reflect the presence of different ligands in different species.

Another important component of innate immunity is the Toll like receptor family. Here, we identified goose TLR3 using our EST library. The extracellular region of goose TLR3 has 22 LRR regions, 1 LRRNT region and 1 LRRCT region (S2 Fig.). The identity of goose TLR3 to other TLRs ranges from 95.88% to 59.45%. Similar to human TLR3, the N-glycosylation sites of goose TLR3 are related to the specific interaction surface structure [30] and are Asn25, Asn43, Asn97, Asn168, Asn219, Asn224, Asn247, Asn360, Asn469, Asn598 and Asn624. Some variant sites are also observed in goose TLR3, such as Asp30, Lys237 and Asp263, which may be specie specific. The conserved disulfide bonds are formed by Cys68-Cys95 and Cys611-Cys639 in goose TLR3. The functional sites, such as Asn219, are important for the response to ds-RNA. Asn168 is related to TLR3 expression levels, and His501 and Asn503 are required for RNA binding and the activation of NF-kappa-B. All of these functional sites are conserved in the goose TLR3 (S2 Fig.).

B-cell activating factor (BAFF) is critical for the stimulation and maturation of B-cells in the adaptive immune system and is also found in our goose cDNA library. The identities among avian species are particularly high and range from 91.99% to 99.65% (S3 Fig.). Similar to the other BAFFs, goose BAFF is mainly composed of a Tumor Necrosis Factor (TNF) domain, which is relatively well conserved among various different species. In the TNF domain, the trimer interface sites are hydrophobic residues such as Gln151, Phe197, Tyr199, Tyr249, Ala254, Tyr281 and Val285 and are conserved from geese to humans. The TNFR 50s-loop binding sites (Leu172, Ser174, Gly212, Lys219 and Ser228 in goose BAFF) are also found in all species without any modification. The conserved long DE loop, known as the “flap”, is unique to BAFF in the TNF family and is located between sites 219 to 228 of goose BAFF. The furin cleavage sites of goose BAFF are “Arg-Gly-Arg-Arg”. Compared with the mammalian BAFFs, the BAFFS are more conserved among the avian species. The Cys235 and Cys248 of goose BAFF are responsible for the formation of conserved intra-chain disulfide bond. The N-Glycosylation site (Asn245) in the TNF domain is conserved among different species, and the other site (Asn102) only found in mouse and human (S3 Fig.). These results indicate that the functional sites of BAFF have changed very little during evolution.

**Fig 9:**
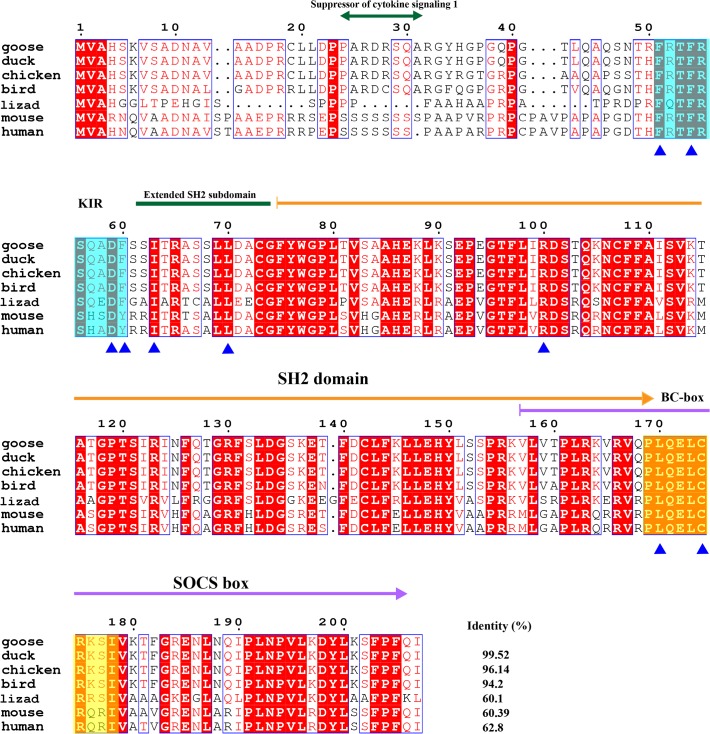
Amino acid alignment of suppressor of cytokine signaling 1 (SOCS1). Amino acid alignment of SOCS1 shows the SH2 domain and SOCS box are respectively indicated by orange arrow and pink arrow. An extended SH2 subdomain is marked with dark green line. Kinase inhibitory region (KIR) is masked with cyan box. The elongin BC complex binding domain, which is also known as BC-box is masked with a yellow box. Suppressor of cytokine signaling region is marked with dark-green double-headed arrow. Functional sites are marked with blue triangles. F51 F54, D59, Y60, I63 and L70 are important for JAK signal transduction suppression and the binding to JH1. R100 is important for LIF signal transduction suppression, the binding to KIT and IL-6 signal transduction suppression. 170L is important for the interaction with elongin BC complex, when it associated with F-179. 174C is also important for the interaction with elongin BC complex, only when it associated with P-175. NCBI accession numbers of SOCS1s are listed as follows: goose: KP238278; duck: 514780149; chicken: 212549671; bird (zebra finch): 224070031; lizard: 637378617; mouse: 409971432; human: 4507233;

**Fig 10:**
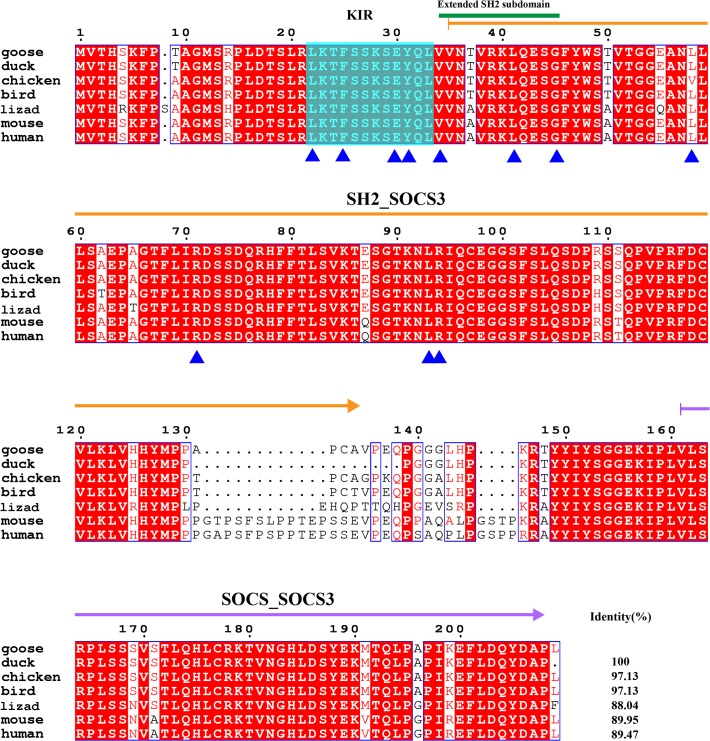
Amino acid alignment of suppressor of cytokine signaling 3 (SOCS3). Amino acid alignment of SOCS3 shows the SH2_SOCS3 domain and SOCS_SOCS3 box are respectively indicated by orange arrow and pink arrow. An extended SH2 subdomain is marked with dark green line. Kinase inhibitory region (KIR) is masked with cyan box. Functional sites are marked with blue triangles. L22, F25, E30, Y31, V34, L41, G45 and R71 have an effect on EPO/LIF-induced signaling suppression. L58, L93 and R94 have an effect on the binding to Y429/Y431 phosphorylated EPOR. NCBI accession numbers of SOCS3s are listed as follows: goose: KP238281; duck: 514711433; chicken: 45382967; bird (zebra finch): 224074414; lizard: 637266413; mouse: 6671758; human: 49168482;

Among the signal transduction pathways, the JAK-STAT pathway is mainly expressed in white blood cells and involved in the regulation of the immune system. Suppressor of cytokine signaling (SOCS) proteins 1 and 3 are inhibitors of JAKs and implicated in inflammation, and they were cloned in this study. Similar to the SOCS proteins from other species, goose SOCS1 is also composed of an SH2 domain and a SOCS box. An extended SH2 subdomain (ESS) is important for JAK phosphotyrosine binding and is located at the beginning of the SH2 domain. The KIR domain is involved in signal and kinase inhibition and is located between Phe51 and Phe60 in goose SOCS1 (Fig. 9). The Elongin BC complex binding domain is known as a BC-box, has the motif (A/P/S/T) -L-x (3) -C-x (3)—(A/I/L/V), and is located between 169 and 179 sites in the goose SOCS1. Compared with the conserved KIR domain and B-C box, the Suppressor of cytokine signaling 1 sequence is 7 or 8 poly-serines and can only be found in mammalian species. The sites for JH1 binding and JAK signal transduction suppression, such as Phe51, Phe54, Asp59, Tyr60, Ile63 and Leu70, are conserved between geese and humans. Arg100, which is important to suppress LIF and IL-6 signal transduction, is highly conserved. According to the alignment, SOCS1 is relatively similar among different species and the identities are all over 60% (Fig. 9). However, SOCS3 is highly conserved; the similarity between goose SOCS3 and duck SOCS3 can reach up to 100% and the smallest similarity is 88.04% (Fig. 10). Similar to SOCS1, the SH2 domain, SOCS box, KIR domain and ESS domain are conserved in goose SOCS3. The sites important for EPO/LIF-induced signaling suppression are Leu22, Phe25, Glu30, Tyr31, Val34, Leu41, Gln45 and Arg71 in goose SOCS3. The Leu58, Leu93 and Arg94 sites of goose SOCS3 are important for the binding to Tyr429/Tyr431 phosphorylated EPOR. These functional amino acids are all conserved from birds to mammals.

The results listed above indicated that the genes involved in adaptive and innate immunity play central roles in goose immunity, while the toll-like receptor, chemokines, complement system and Jak-STAT signaling pathway act as the functional bridge between the innate and adaptive immune responses.

### STRING Analysis

**Fig 11:**
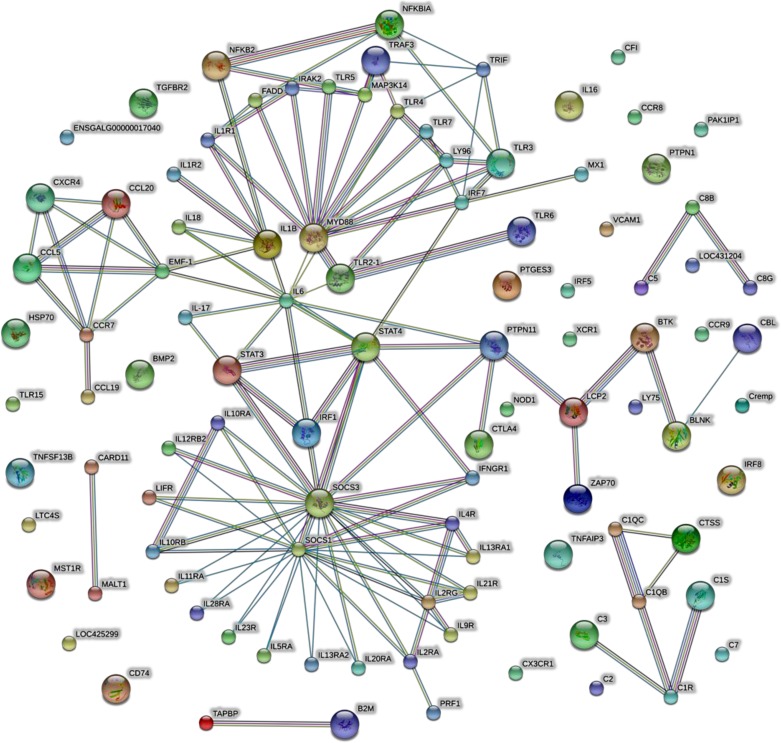
STRING analysis of immune relevant genes from the goose peripheral blood lymphocytes transcriptome. The network nodes represent the proteins encoded by the DE genes. Seven different colored lines link a number of nodes and represent seven types of evidence used in predicting the associations. A red line indicates the presence of fusion evidence; a green line represents neighborhood evidence; a blue line represents co-occurrence evidence; a purple line represents experimental evidence; a yellow line represents text-mining evidence; a light blue line represents database evidence, and a black line represents co-expression evidence.

Based on the identified 125 immune-related genes, STRING 9.1 (http://string-db.org/) was used to analyze the interactions and relationships among these genes. From 125 goose genes, 105 were matched well to the known immune genes of *Gallus gallus* in the STRING database (Fig. 11). In particular, the genes, whose sequence information has been confirmed by our PCR reactions, play important roles in the immune interaction nets. Like SOCS3, it can interact with IL receptors of Cytokine-cytokine receptor interaction pathway, STATs of Jak-STAT signaling pathway, PTPN11 of Natural killer cell mediated cytotoxicity pathway and IRF1 of Transcription factors for immune response pathway. By the interaction of SOCS3, various immune pathways are connected as a whole one and SOCS1, SOCS3 and PTPN11 were very strongly linked with many signaling pathways, which indicates that all of these genes interacted with each other and comprise a classical network of different genes. C1qs are also the key knots for complement pathway by interact with C1s and C1r and then in the downstream, C3 was activated. Myd88 is the centre modular of Toll-like receptor signaling pathway. It can interact with various TLRs, including TLR3, leading to NF-kappa-B activation of immune defense. No connection is observed in BAFF/TNFFSF13B, it may due to the absence of BAFF receptors in this analysis. These results show us these immune genes of goose have similar functions and reaction modes as in other species (like chicken). These immune molecules do not work independently and they can function in various pathways to link the immune system as a whole one for the immune defense.

## Discussion

As a source of meat, eggs and feathers, the goose is one of the most important economical waterfowl around the world. As geese serve as one of the principal natural reservoirs for influenza A viruses, its study is of special interest in medicine and public health problem [31,32]. Due to lack of genomic information, it is important for us to understand the immune repertoires of geese. After the high-throughput RNA sequencing of the transcriptome, it became one of the most convenient method to obtain the overall gene information. Here, we present the study about the immune-related genes and pathways in goose transcriptome. The results describe the genetic architecture of the goose PBLs transcriptome and further explore their relevant immune relevant genes.

In this study, we pooled RNA from normal healthy geese and performed deep sequencing using the Illumina platform. This pooling strategy was widely used in other similar studies [2, 14, 15]. To date, only 2 studies have reported on the transcriptome data from goose ovaries and other body tissues [2–15]. In this study, a large genomic description of goose PBLs was provided. Compared to other transcriptome data, this goose PBLs transcriptome library is larger in data size and has more raw reads (91.93 million compared to 84.14 million and 4.36 million), more clean mapped reads (69.36 million compared to 60.50 million and 3.70 million), and more total annotated matched unigenes (211,198 compared to 568, 39 and 130,517) [2–15], which characterize the precise gene expression in different tissues in contrast to the goose PBLs. These data can provide useful information for further investigations of the goose genome.

We generated 211,198 unigenes for 69.5 MB total length of the goose PBLs transcriptome. The overall GC content of the transcriptome was calculated to be 47%, which closely resembles the percent GC content that was reported in a previous study [14]. The size distribution indicated that the length of the 91,069 unigenes was more than 1500 bp, which is much higher than that reported by other researchers [2, 15] for the goose transcriptome. We also noticed that the mean length of unigenes was longer than that reported in other research [2, 15]. We compared our unigenes against the NCBI Nr protein database, allowing further functional annotation and classification using GO, KOG and KEGG. This functional annotation provides expected information on biological function and biosynthesis pathways for assembled unigenes. We also found that the Nr database had maximum annotated unigenes and most of the aligned sequences matched with the chicken (17,238, 55.59%) sequence, suggesting that more genetic similarities exists between the goose (a waterfowl bird) and chickens (a domestic bird). Among the Nr blast hits, only 128 genes were matched to the goose itself, illustrating the limited number of the goose related genes currently available in the NCBI database. We also found that 26,985 (73.2%) of the unigenes identified in this study have high similarity to the NCBI blast result (Fig. 1E) and are covered by 80–100% in the length, which supports the validity of our transcriptome data.

The GO annotation shows that a large number of the unigenes are in the biological processes (72,844) category. As expected, many genes which are involved in the defense system of geese, including response to stimulus (7,075 unigenes), signaling (3,570), and immune system process (512), were found in this study. We identified a large number of unigenes that are involved in the immune system (2,757) in the KEGG pathway’s organismal systems category to further elucidate the biological functions of these genes in geese. In our goose PBLs transcriptome data, we found that a large number of the unigenes were in the immune relevant pathways and were involved in well-recognized immune pathways, such as chemokine signaling pathway 348, B-cell receptor, T-cell receptor 134, Toll-like receptor signaling pathway 204, leukocyte transendothelial migration 244, antigen processing and presentation 74 (Fig. 4). The availability of these data would provide an abundant resource for understanding the pathways of the goose immune system. Additionally, among the 2,757 unigenes that participated in 15 immune system KEGG pathways, we focused on the 125 most important novel genes of the immune system of goose that were identified. We observed that 109 unigenes of the 125 unigenes described here, are novel genes in the goose that were first identified in this study.

Our transcriptome sequence revealed the presence of immune relevant genes in goose. There were no evidence existed, in that majority of the detected immune related genes in goose before performing this PBLs transcriptome analysis. Here, we are the first to identify several important genes of the immune pathways and to compare them with genes in the duck, chicken, turkey and zebra finch. The genomic information has been made available in the NCBI database to facilitate the detection of novel genes in the goose (Fig. 5/ Table 4). Except for the duck, the other avian species are different in both habitat and avian family from the goose. Using the NCBI database, we found that 104 of the 125 unigenes in the goose PBLs transcriptome were shared with the duck (a waterfowl like the goose) and 21 genes (including TLR13, CC26, IL8, IFR3, IFR7) were not shared. In the NCBI database, 8 genes (including CCL14, CCL26, IFR3, C4–1) were not shared in the chicken, 43 genes (including TAPBP, HSP70, HSP90A, TLR3, TLR13, LY96, C8G, BAFF) were not found in the turkey and 30 genes (including TAPBP, TLR13, TLR15, CCL14, CCL19, IL9R, IL23R, C2, MBL) were not found in zebra finch. A comparison of our sequencing results with the information available in the NCBI databases for four other species provides more genomic and transcriptomic information and can contribute to the study of the avian transcriptome.

Our ongoing studies on these genes may extend the list of variations in the immune genes of goose with other species. Here, we describe the 10 most likely putative genes of the innate, adaptive immune system and signaling pathways identified in the goose PBLs transcriptome, which were confirmed by PCR and further by comparison of their sequences to that of other species.

In the complement system, C1qA, C1qB and C1qC were cloned and further analyzed by comparison. According to the comparative analysis, goose C1qA, C1qB and C1qC all show the highest identity to the corresponding genes in the duck. The goose is most closely evolutionarily related to the duck and that was further confirmed by an evolutionary tree (S4 Fig.). In the tree, the evolutionary distance between different species are arranged consistent with time of emergence of each species. As C1qA, C1qB and C1qC belong to the same C1q family, they have a common evolutionary origin in the evolutionary tree. The molecule architecture and functional sites in the collagen-like domains of these three molecules are conserved among birds, reptiles and mammals. However, the receptor binding sites in the C1q domains varied considerably in comparison to other species. We also cloned complement component 8, gamma polypeptide (C8G), which is a constituent of the membrane attack complex. The C8G alignment results show conserved characteristics among the different species (S5 Fig.). With the exception of some functional studies [33], little is known about the avian toll-like receptor pathway, especially TLR3. Here, the goose TLR3 is shown to have conserved domains and sites with the human TLR3, indicating its evolutionary conservation. Overall, 5 genes of the innate immune system, including C1qA, C1qB, C1qC, C8G and TLR3, have been identified in the goose.

Studies have shown that goose BAFF is a conserved molecule in the adaptive immune system and that it is able to promote bursal B cell survival and proliferation in the goose [34]. Here, we further confirm the BAFF sequence in our goose cDNA library. BAFF is conserved among the avian species, but diverges from the mammalian gene. CD74, the MHCII invariant chain, play critical roles in MHC class II antigen processing by stabilizing peptide-free MHCII heterodimers and was also cloned in this study [10]. The sequence of the goose CD74 clone here is similar to the one submitted to NCBI, which indicates its functional and conservational importance. IL1RL1/ST2, an IL-1 family cytokine that can activate NF-κB and MAP kinases and drive production of TH2-associated cytokines from T helper type 2 cells [35], play important roles in both the innate and adaptive immune systems. The IL1RL1 gene of goose was also identified in our study. IL1RL1 has 3 Ig-like C2 domains and one TIR domain. Of the 5 mammalian disulfide bonds, 4 are found to be conserved at the Cys32-Cys83, Cys109-Cys145, Cys128-Cys175 and Cys228-Cys295 sites in the goose IL1RL1 (S6 Fig.). According to the alignment, the IL1RL1s in avian species share more sequence similarity with those in reptiles than with those in mammals. For instance, the positions of the N- Glycosylation sites are not well conserved between the goose and human IL1RL1s.

JAK-STAT pathway plays a central role in lots of biological processes of both innate and adaptive immunity. SOCS family proteins are part of a classical negative feedback system that regulates cytokine signal transduction. SOCS3 and SOCS1 are negative regulators of cytokines that signal through the JAK/STAT pathway. Here, SOCS1 and SOCS3 genes have been cloned from the goose and shown to have conserved structural and functional sites. The evolutionary tree indicates that SOCS1 and SOCS3 share a common origin (S7 Fig.). Over long evolutionary distances, the avian SOCS1s diverged after the split between the reptile, avian and mammalian lineage. In the evolutionary tree, the SOCS3s of different species are clustered together, indicating that little change occurred over the course of evolution.

The 125 immune related genes were further analyzed by gene interaction networks (STRING analysis). The results show us a similar immune response network as in other avian species, and it also confirms the potential functions of these genes in the goose.

## Conclusion

The data described here provide the first PBLs transcriptome profile of the goose immune system. Among 211,198 unigenes, 2,757 unigenes of immune system, 17 immune related pathways and their unigenes, 125 important immune genes have been found in the goose EST and compared between the goose, duck, chicken, turkey and zebra finch. The 10 most important immune genes of the goose have been cloned and analyzed. This information will give us an overall landscape of the goose immune system and assist us in understanding the goose immune system. We believe that the availability of this annotated transcriptome will facilitate the isolation and characterization of the functional genes involved in different immune system pathways, as well as validate the molecular genetic approach to disclose the immune system of goose.

## Supporting Information

S1 Fig PCR Validation of the goose immune related genes. PCR confirmation of 10 immune related genes expression (BAFF, C1qA, C1qB, C1qC, CD74, C8G, SOCS1, SOCS3, IL1RL1, and TLR3) from the peripheral blood lymphocytes of goose and analyzed by gel electrophoresis.
(TIF)

S2 Fig Amino acids alignment of Toll-like Receptor 3 (TLR3). Amino acid alignment of TLR3 shows that the 22 LRR regions are indicated with green arrows. LRRNT and LRRCT are indicated with orange arrows. Cystines, which forms inter-chain disulfide bonds, are masked with yellow boxes. Glycosylation sites are marked with blue triangles. Functional sites are marked with blue triangles. C68, 95C and 219N are important for the response to ds-RNA. 168N is related to its expression levels. 501H and 503N are important for RNA binding and activation of NF-kappa-B. NCBI accession numbers of TLR3s are listed as follows: goose: KP238287; duck: 705772385; chicken: 119394689; bird (zebra finch): 224049815; lizard: 637306366; mouse: 71534005; human: 86161330;
(TIF)

S3 Fig Amino acids alignment of B-cell activation factor (BAFF). BAFF Amino acid alignment indicates the cytoplasmic, transmembrane and extracellular regions are marked respectively. TNF domain region is indicated with green arrow. N-Glycosylation sites are indicated by hollow blue triangles. Cystines involved in intra-chain disulfide bond are masked by yellow rectangles. Residues in cyan rectangle are in the formation of Trimer interface 7. Magenta pentacle indicates receptor binding sites5. Residues masked by green rectangle are the conserved long DE loop, known as “flap”. Residues masked by purple rectangle are the conserved furin cleavage sites. NCBI accession numbers of BAFFs are listed as follows: goose: KP238285; goose-publish: 114159808; duck: 90025061; chicken: 32815310; guail: 193090153; alligator: 296399288; mouse: 13124571; human: 13124573.
(TIF)

S4 Fig Evolutionary tree of C1qA, C1qB and C1qC. Evolutionary tree based on the alignment of amino acid sequences from three proteins (C1qA, C1qB and C1qC) of Chinese goose with those of other species was constructed by the neighbor-joining method with Mega 5.1 software. The evolutionary distance among different species is arranged consistent with emergence times of these species. As C1qA, C1qB and C1qC belonging to the same C1q family, they have a common evolutionary origin in the evolutionary tree. The numbers near the branches are bootstrap percentages supporting the given branching pattern. Branch lengths are measured in terms of amino acid substitutions, with scale indicated below the trees.
(TIF)

S5 Fig Amino acids alignment of complement component 8, gamma (C8G). Amino acid alignment of complement component 8, gamma polypeptide (C8G), which is a constituent of the membrane attack complex and it shows conserved characteristics among different species. The goose identity with other species is listed at the end. NCBI accession numbers of C8Gs are listed as follows: goose: KP238282; duck: 514725119; chicken: 363740281; falcon: 541979148; lizard: 637368602; mouse: 422010931; human: 119608722;
(TIF)

S6 Fig Amino acids alignment of Interleukin 1 receptor type 1 (IL1RL1). Three Ig like C2 domains are indicated by green arrows. TIR domain is marked with yellow arrow. Cystines, which forms inter-chain disulfide bonds, are masked with yellow boxes. Glycosylation sites are marked with blue triangles. Cytoplasmic region, extracellular region and transmembrane are indicated respectively. NCBI accession numbers of IL1RL1s are listed as follows: goose: KP238283; duck: 514719303; chicken: 66954656; bird (zebra finch): 224042933; alligator: 557298620; mouse: 30410944; human: 21411306;
(TIF)

S7 Fig Evolutionary tree of SOCS1 and SOCS3. Evolutionary tree based on the alignment of amino acid sequences from SOCS1 and SOCS3 of Chinese goose with those of other species was constructed by the neighbor-joining method with Mega 5.1 software. The evolutionary tree indicates a common origin of SOCS1 and SOCS3. The numbers near the branches are bootstrap percentages supporting the given branching pattern. Branch lengths are measured in terms of amino acid substitutions, with scale indicated below the trees.
(TIF)

S1 Table Genes and specific primers used for PCR. Ten genes were selected based on their functions in innate, adaptive immune system and signaling pathways. Primer sequences were designed according to sequences from our transcriptome data of goose PBLs.
(PDF)
