# Supplementary figures and images for: De Novo Transcriptomic Analysis of Peripheral Blood Lymphocytes from the Chinese Goose: Gene Discovery and Immune System Pathway Description
Source: PLoS One. 2015 Mar 27;10(3):e0121015. doi: 10.1371/journal.pone.0121015 (PMC4376690; doi:10.1371/journal.pone.0121015)

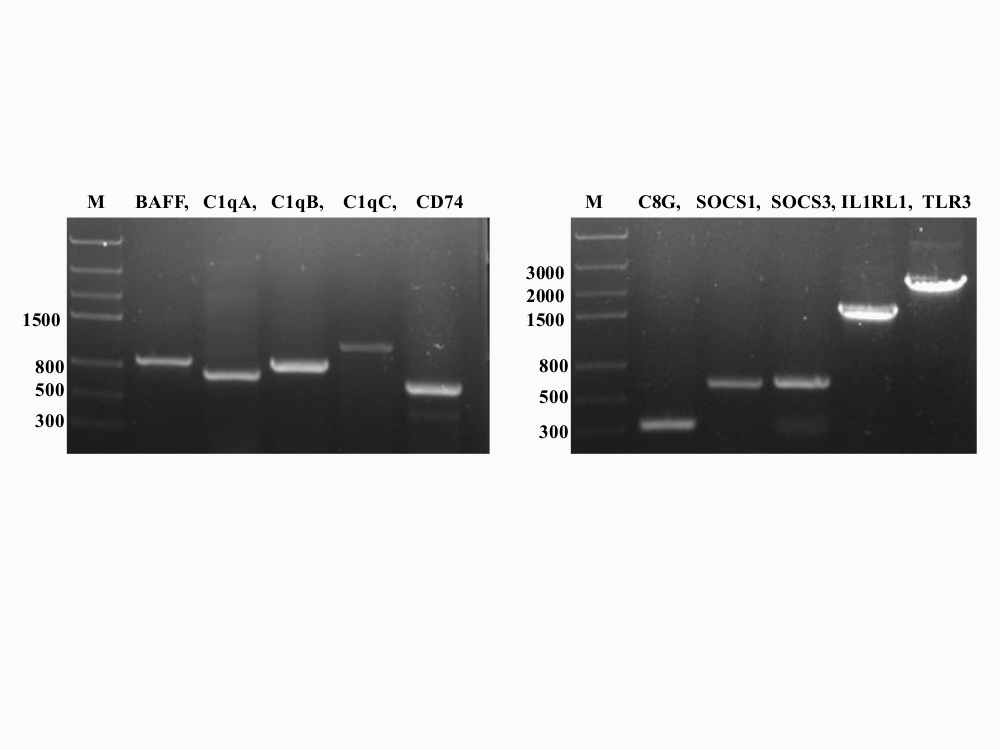

Supplement: S1 Fig — PCR confirmation of 10 immune related genes expression (BAFF, C1qA, C1qB, C1qC, CD74, C8G, SOCS1, SOCS3, IL1RL1, and TLR3) from the peripheral blood lymphocytes of goose and analyzed by gel electrophoresis. (TIF) [file pone.0121015.s001.tif]

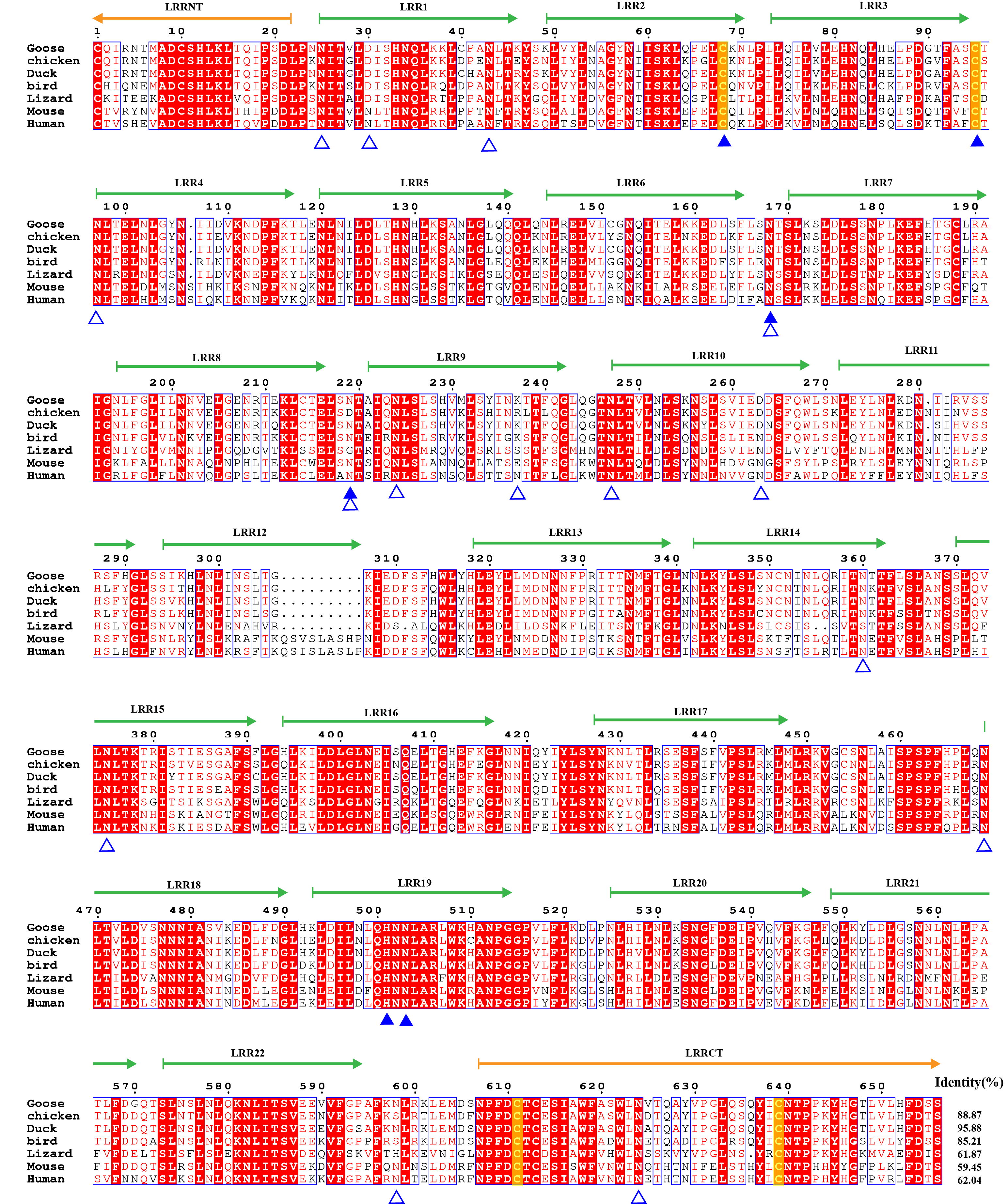

Supplement: S2 Fig — Amino acid alignment of TLR3 shows that the 22 LRR regions are indicated with green arrows. LRRNT and LRRCT are indicated with orange arrows. Cystines, which forms inter-chain disulfide bonds, are masked with yellow boxes. Glycosylation sites are marked with blue triangles. Functional sites are marked with blue triangles. C68, 95C and 219N are important for the response to ds-RNA. 168N is related to its expression levels. 501H and 503N are important for RNA binding and activation of NF-kappa-B. NCBI accession numbers of TLR3s are listed as follows: goose: KP238287; duck: 705772385; chicken: 119394689; bird (zebra finch): 224049815; lizard: 637306366; mouse: 71534005; human: 86161330; (TIF) [file pone.0121015.s002.tif]

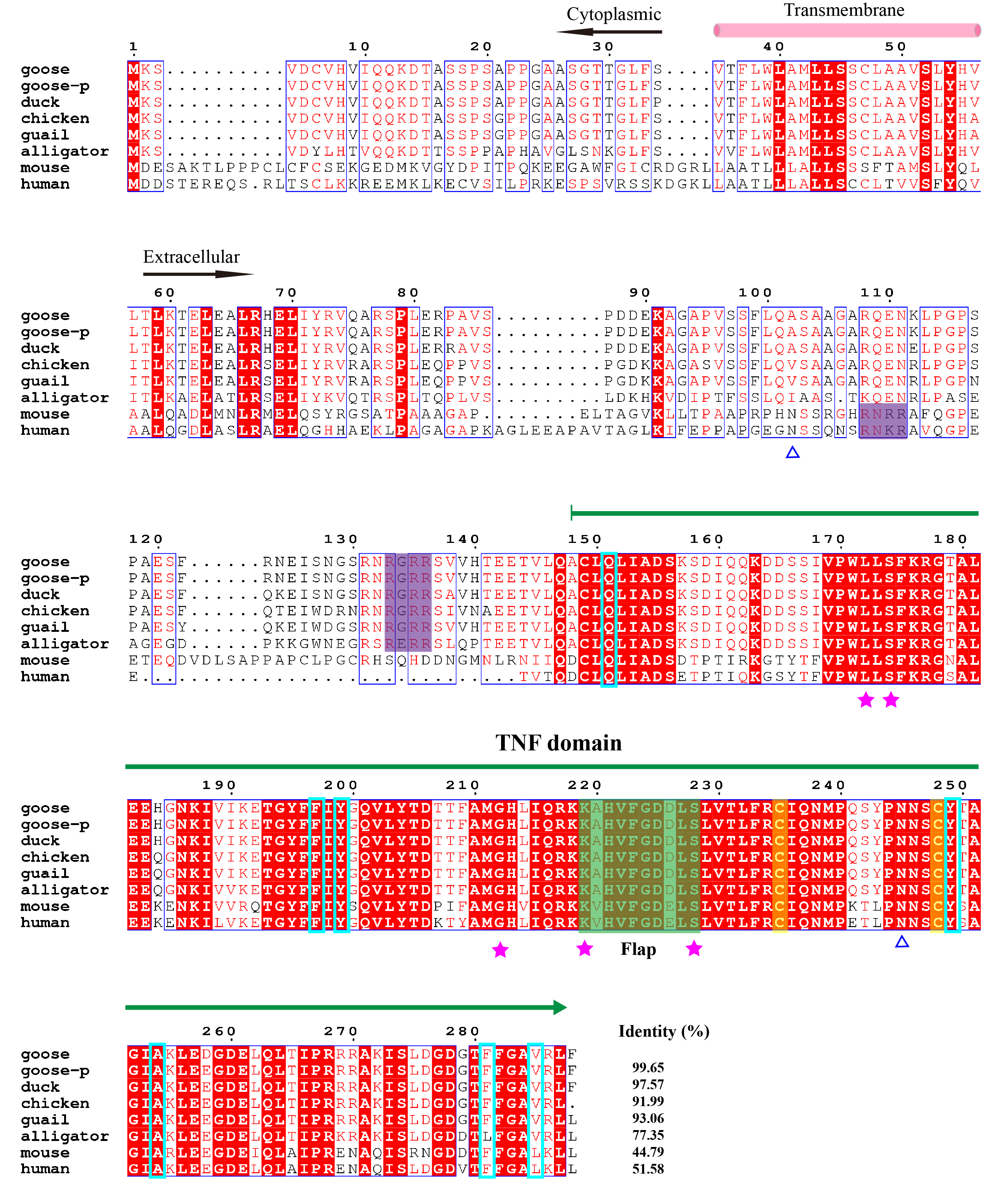

Supplement: S3 Fig — BAFF Amino acid alignment indicates the cytoplasmic, transmembrane and extracellular regions are marked respectively. TNF domain region is indicated with green arrow. N-Glycosylation sites are indicated by hollow blue triangles. Cystines involved in intra-chain disulfide bond are masked by yellow rectangles. Residues in cyan rectangle are in the formation of Trimer interface 7. Magenta pentacle indicates receptor binding sites5. Residues masked by green rectangle are the conserved long DE loop, known as “flap”. Residues masked by purple rectangle are the conserved furin cleavage sites. NCBI accession numbers of BAFFs are listed as follows: goose: KP238285; goose-publish: 114159808; duck: 90025061; chicken: 32815310; guail: 193090153; alligator: 296399288; mouse: 13124571; human: 13124573. (TIF) [file pone.0121015.s003.tif]

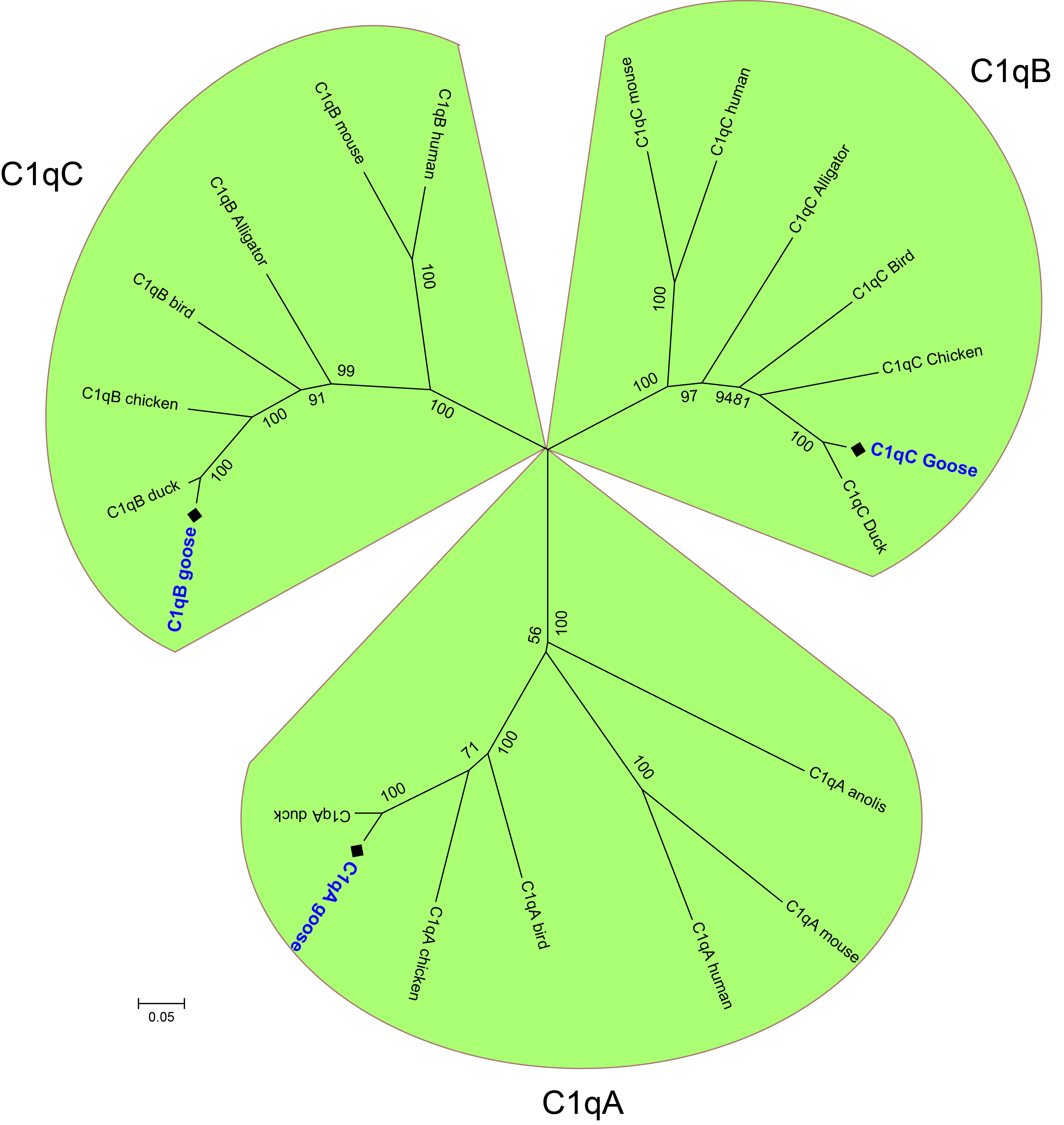

Supplement: S4 Fig — Evolutionary tree based on the alignment of amino acid sequences from three proteins (C1qA, C1qB and C1qC) of Chinese goose with those of other species was constructed by the neighbor-joining method with Mega 5.1 software. The evolutionary distance among different species is arranged consistent with emergence times of these species. As C1qA, C1qB and C1qC belonging to the same C1q family, they have a common evolutionary origin in the evolutionary tree. The numbers near the branches are bootstrap percentages supporting the given branching pattern. Branch lengths are measured in terms of amino acid substitutions, with scale indicated below the trees. (TIF) [file pone.0121015.s004.tif]

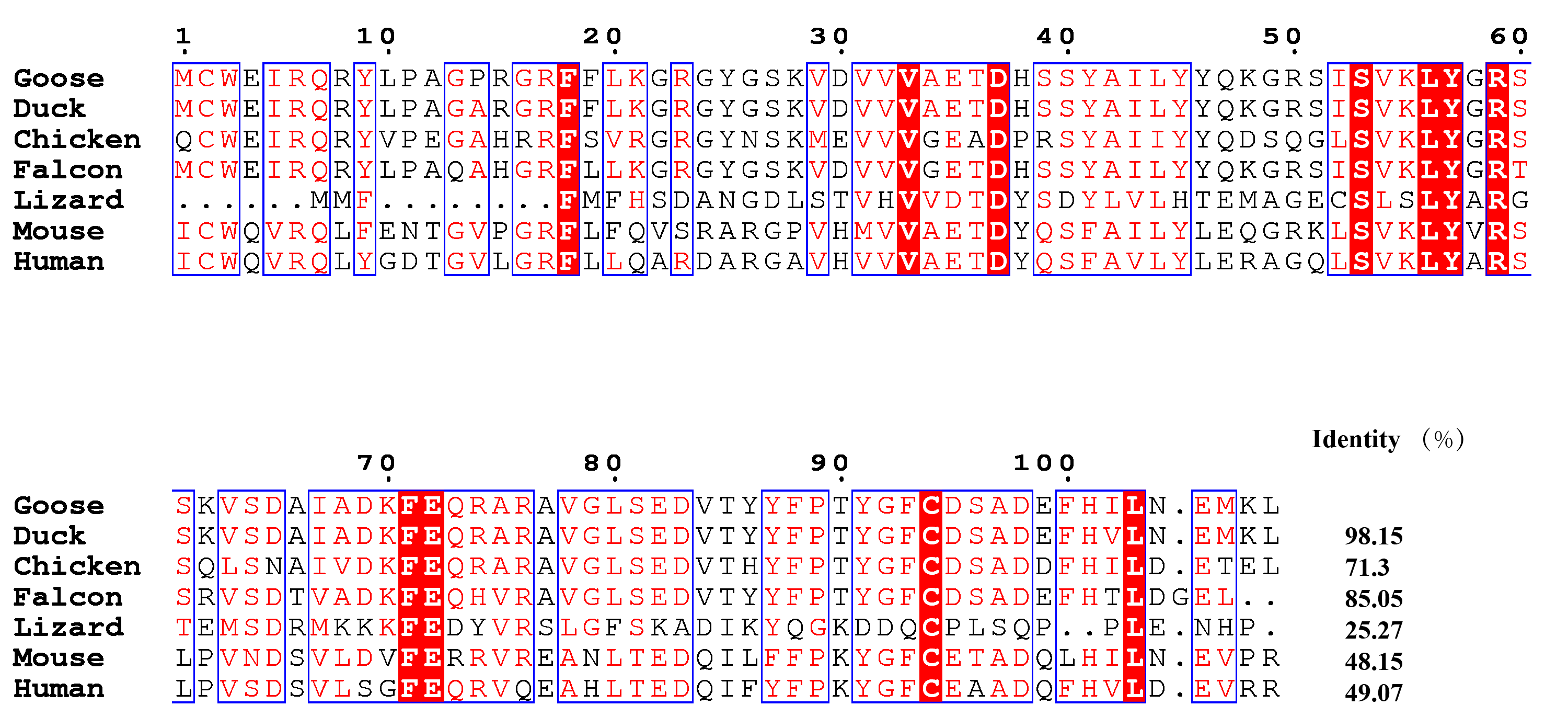

Supplement: S5 Fig — Amino acid alignment of complement component 8, gamma polypeptide (C8G), which is a constituent of the membrane attack complex and it shows conserved characteristics among different species. The goose identity with other species is listed at the end. NCBI accession numbers of C8Gs are listed as follows: goose: KP238282; duck: 514725119; chicken: 363740281; falcon: 541979148; lizard: 637368602; mouse: 422010931; human: 119608722; (TIF) [file pone.0121015.s005.tif]

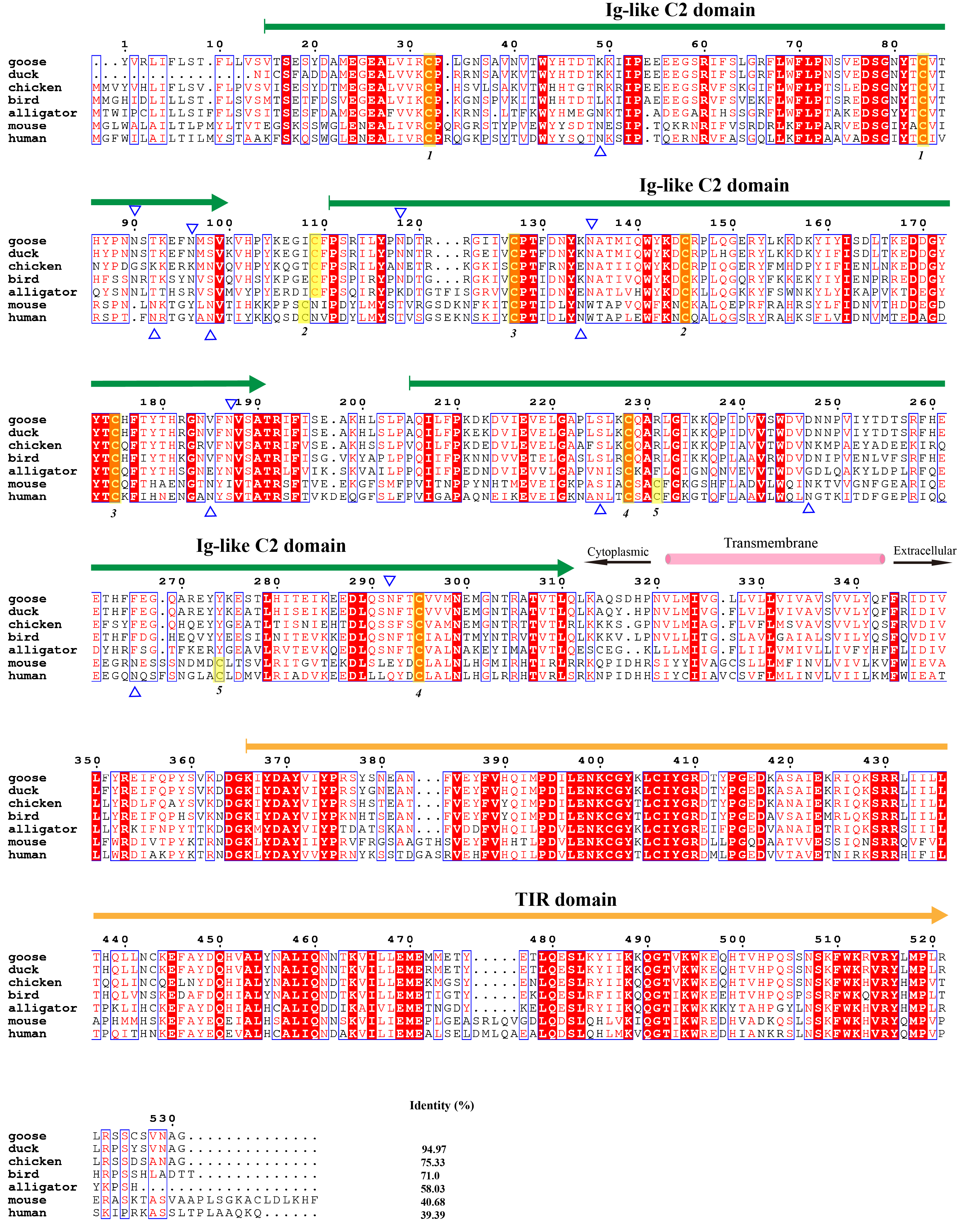

Supplement: S6 Fig — Three Ig like C2 domains are indicated by green arrows. TIR domain is marked with yellow arrow. Cystines, which forms inter-chain disulfide bonds, are masked with yellow boxes. Glycosylation sites are marked with blue triangles. Cytoplasmic region, extracellular region and transmembrane are indicated respectively. NCBI accession numbers of IL1RL1s are listed as follows: goose: KP238283; duck: 514719303; chicken: 66954656; bird (zebra finch): 224042933; alligator: 557298620; mouse: 30410944; human: 21411306; (TIF) [file pone.0121015.s006.tif]

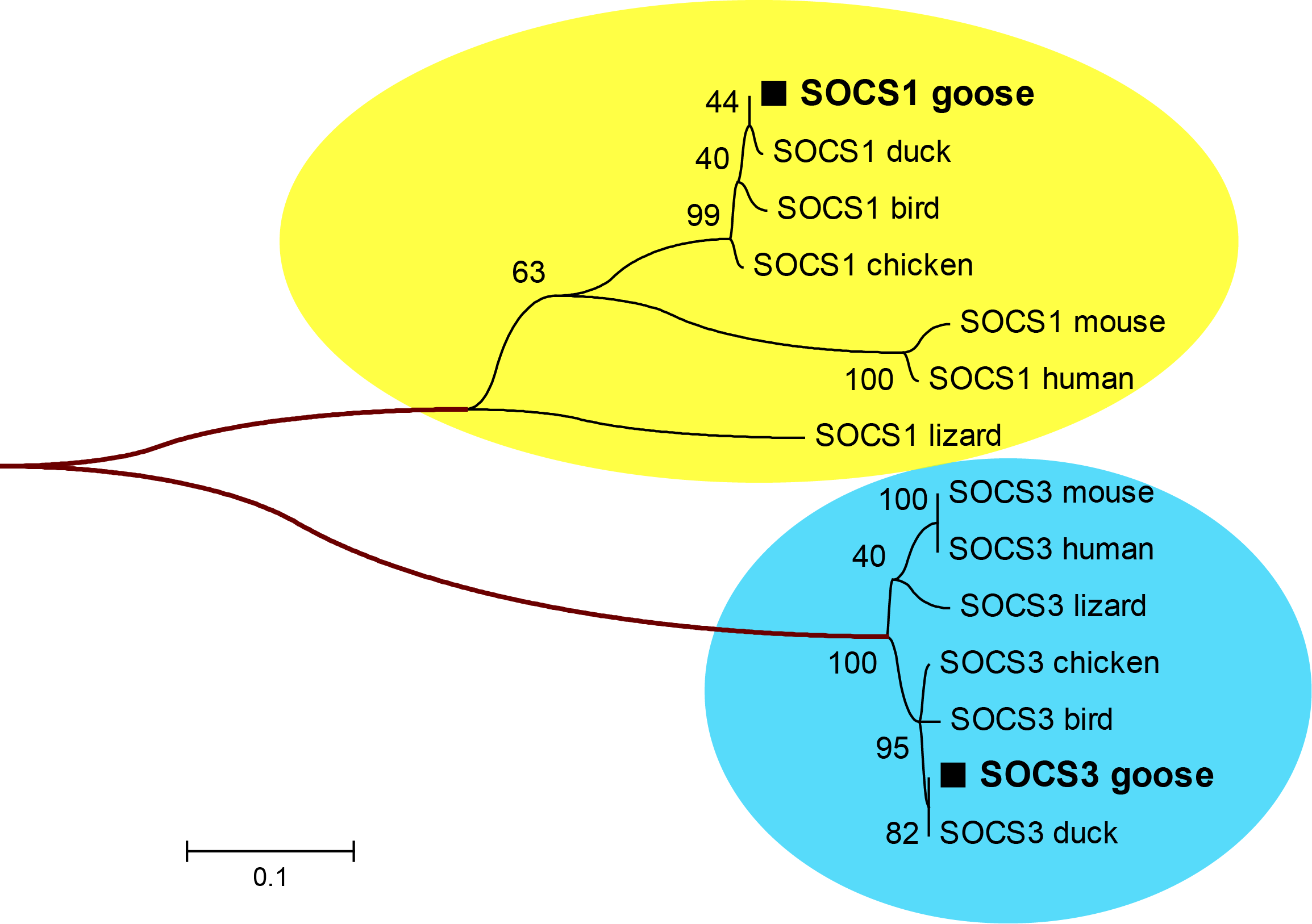

Supplement: S7 Fig — Evolutionary tree based on the alignment of amino acid sequences from SOCS1 and SOCS3 of Chinese goose with those of other species was constructed by the neighbor-joining method with Mega 5.1 software. The evolutionary tree indicates a common origin of SOCS1 and SOCS3. The numbers near the branches are bootstrap percentages supporting the given branching pattern. Branch lengths are measured in terms of amino acid substitutions, with scale indicated below the trees. (TIF) [file pone.0121015.s007.tif]
