# Supplementary material for: De Novo Transcriptomic Analysis of Peripheral Blood Lymphocytes from the Chinese Goose: Gene Discovery and Immune System Pathway Description
Source: PLoS One. 2015 Mar 27;10(3):e0121015. doi: 10.1371/journal.pone.0121015 (PMC4376690; doi:10.1371/journal.pone.0121015)
Supplement: S1 Table — Ten genes were selected based on their functions in innate, adaptive immune system and signaling pathways. Primer sequences were designed according to sequences from our transcriptome data of goose PBLs. (PDF) [file pone.0121015.s008.pdf]

S1 Table. Genes and specific primers used for PCR. Ten genes were selected based on their functions in innate, adaptive immune system and signaling pathways. Primer sequences were designed according to sequences from our transcriptome data of goose PBLs.

Gene Name	Primer sequence (5'--3')	Gene size
B-Cell activating factor	F-ATGAAATCCGTGGACTGTGT	867bp
	R-TCAGAAGAGTCTGACTGCAC	
Complement C1q subcomponent subunit A	F-ATGCGTCTCGGTCTTTGGCTGG	732bp
	R-TCAGCCCGTCTGTGGGAAGAGC	
Complement C1q subcomponent subunit B	F-ATGTGGCCAGATTCTTTCACAAC	906bp
	R-CTAAGCCTCAGGGAAGATCAGG	
Complement C1q subcomponent subunit C	F-CCTATCGGGCTCTTTCCTAACT	920bp
	R- AGAGCAGGAACCCCGAGAA	
Complement component C8 gamma chain	F-ATGTGCTGGGAGATCAGGCAG	327bp
	R- CTAGAGCTTCATTTCGTTGAG	
CD74 molecule, MHC class II associated invariant chain.	F-ATGGCCGAGGAGCAGCGGGACCTC	670bp
	R- CTACTTGGCTTTGTCGGCGCCCAG	
Interleukin 1 receptor type 1	F-GGTATGTACGTTTGATATTCC	1598bp
	R- TCATCCAGCATTAACTGAGC	
Suppressor of cytokine signalling 1	F- ATGGTAGCGCACAGCAAGGTG	624bp
	R- TTAGATCTGAAATGGGAAGGA	
Suppressor of cytokine signalling 3	F- ATGGTCACCCACAGCAAGTTC	630bp
	R- TTAGAGGGGGGCATCGTACTG	
Toll-like receptor 3	F- GATGGTTTTTGTGCACTGA	2386bp
	R- CCAGAGAGATGAAATTCTT	
